# Machine Learning-Based Prediction of Antimicrobial Resistance in *Escherichia coli* from MALDI-TOF Mass Spectrometry Data

**DOI:** 10.3390/diagnostics16132103

**Published:** 2026-07-04

**Authors:** Nick Versmessen, Marieke Mispelaere, Robin Vanstokstraeten, Mariana Teixeira, Jerina Boelens, Cedric Hermans, Marjolein Vandekerckhove, Katleen Vranckx, Paco Hulpiau, Thomas Demuyser, Sven Degroeve, Piet Cools

**Affiliations:** 1 Department of Diagnostic Sciences, Faculty of Medicine and Health Sciences, Ghent University, C. Heymanslaan 10, 9000 Ghent, Belgium; 2 Bioinformatics Knowledge Center (BiKC), Cluster Life Sciences, Howest University of Applied Sciences, Spoorwegstraat 4, 8200 Brugge, Belgium; 3 Department of Microbiology and Infection Control, University Hospital of Brussels (UZ Brussel), Vrije Universiteit Brussel (VUB), Laarbeeklaan 101, 1090 Brussels, Belgium; 4 Polytechnic Institute of Coimbra, School of Health Technology of Coimbra, Rua Da Misericórdia, Lagar Dos Cortiços, S. Martinho Do Bispo, 3045-093 Coimbra, Portugal; 5 Department of Medical Microbiology, Ghent University Hospital (UZ Gent), 9000 Ghent, Belgium; 6 Augmented Diagnostics, Industrial Applications, BioMérieux, 1030 Brussels, Belgium; 7 Department of Medical Microbiology, University Hospital of Antwerp (UZ Antwerpen), Drie Eikenstraat 655, 2650 Edegem, Belgium; 8 AIMS Lab, Center for Neurosciences, Vrije Universiteit Brussel (VUB), 1000 Brussels, Belgium; 9 Laboratory of Applied Microbiology and Biotechnology (LAMB), University of Antwerp, 2000 Antwerp, Belgium; 10 VIB-UGent Center for Medical Biotechnology, VIB, a. Baertsoenkaai 3, 9000 Ghent, Belgium; 11 Department of Biomolecular Medicine, Ghent University, 9000 Ghent, Belgium

**Keywords:** antibiotic resistance, machine learning, MALDI-TOF MS

## Abstract

**Objectives:** To assess the feasibility and reproducibility of predicting antimicrobial resistance (AMR) in *Escherichia coli* from MALDI-TOF mass spectrometry data using a standardized, open-source machine learning (ML) workflow, we systematically compared four ML algorithms, evaluated the impact of culture conditions, extract storage, and spectral preprocessing on model performance, and validated results through nested cross-validation with statistical significance testing. **Methods:** A total of 282 clinical *E. coli* isolates were analyzed. Two MALDI-TOF MS datasets were generated from freshly cultured extracts (T_1_) and recultured isolates one year later (T_3_), yielding 4468 spectra. A third dataset from the T_1_ extracts stored at −20 °C for one year (T_2_) was evaluated for spectral stability but excluded from primary modeling likely due to storage-induced degradation. Protein spectra (*m*/*z* 2000–15,000) were preprocessed using an in-house developed MALDI-TOF preprocessing pipeline (MTPP) comprising variance stabilization, Savitzky–Golay smoothing, SNIP baseline correction, TIC normalization, LOWESS alignment, and MAD-based peak detection (SNR ≥ 3), yielding 121 *m*/*z* features. Four classifiers—Random Forest (RF), Logistic Regression, Support Vector Machine, and Gradient Boosting—were trained to predict resistance to 11 antibiotics using nested cross-validation: outer GroupShuffleSplit (5-fold, isolate-level) for evaluation and inner GroupKFold for recursive feature elimination (RFECV) and hyperparameter tuning (RandomizedSearchCV). Classification thresholds were optimized via the precision–recall curve. Model performance was assessed using AUROC, AUPRC, F1-score, Matthews Correlation Coefficient (MCC), and bootstrap 95% confidence intervals (1000 replicates). Pairwise model comparisons were tested with McNemar’s chi-squared test. **Results:** Among the 12 antibiotics included in the analysis (meropenem excluded for absence of resistance), resistance prevalence ranged from 1.1% (colistin) to 59.9% (amoxicillin). Colistin was subsequently also excluded from ML modeling due to insufficient resistant isolates (*n* = 3), leaving 11 antibiotics for prediction. The best predictive performance was observed for ciprofloxacin (AUROC 0.76 [95% CI 0.74–0.77]; F1 0.54; MCC 0.38) and ceftazidime (AUROC 0.68 [0.65–0.71]; F1 0.36; MCC 0.29), using 13 and 37 RFECV-selected features, respectively. Amoxicillin achieved the highest F1-score (0.76), driven by high recall (0.98) but modest AUROC (0.58). No meaningful predictive signal was detected for amikacin, cefepime, or tigecycline (AUROC ≤ 0.57, F1 ≤ 0.17), attributable to extreme class imbalance, and no robust multi-peak resistance signature was detected in this dataset. McNemar’s test confirmed that RF significantly outperformed Logistic Regression for all antibiotics (*p* < 0.01), while Gradient Boosting performed comparably to RF for ciprofloxacin (*p* = 0.17) and ceftazidime (*p* = 0.28). Frozen extracts (T_2_) produced lower spectral similarity and were excluded from model training; the aligned T_1+3_ dataset yielded the most stable performance across metrics. **Conclusions:** Machine learning analysis of MALDI-TOF spectra enables reproducible AMR prediction for selected antibiotics in *E. coli*, with ciprofloxacin and ceftazidime showing the strongest signal. Nested isolate-level cross-validation, multi-model comparison with statistical testing, and open-source code provide a transparent, reproducible foundation for integrating ML-assisted MALDI-TOF analysis into diagnostic AMR surveillance. Extract storage at −20 °C degrades spectral quality and should be avoided in ML training workflows.

## 1. Introduction

Matrix-assisted laser desorption ionization time-of-flight mass spectrometry (MALDI-TOF MS) was introduced into clinical microbiology laboratories in 2009 and has since become a standard tool for the identification of bacterial and yeast isolates [1,2,3]. The identification process involves treating biological material derived from isolates with an ionization matrix and subjecting the sample to laser desorption/ionization to generate peptide mass fingerprints (PMFs), which are distinctive spectral profiles that represent peptide peak patterns unique to each species. Identification is achieved by matching these PMFs with entries in reference databases [1]. Species-level identification is a first step to guide clinicians in selecting appropriate antimicrobial treatment [1,2].

Depending on the species and clinical context, selected isolates are further tested for antimicrobial resistance, a core task in clinical microbiology. Conventional methods, such as broth microdilution to determine the minimum inhibitory concentration (MIC) or the Kirby–Bauer disk diffusion test, remain the gold standard [4]. These methods require overnight culturing, meaning that although results are typically available within one to two days, they are often not read or reported until the following day. As a result, antimicrobial therapy is frequently initiated empirically before susceptibility data are available, with adjustments to the therapeutic regimen made once the results are reviewed [4,5].

Although these tools are highly reliable, diagnostic workflows can still face practical challenges, including high sample volumes, time constraints, and reliance on specialized personnel [6,7,8]. The integration of artificial intelligence (AI) and machine learning (ML) into diagnostic workflows holds promise for overcoming several of these limitations, offering new opportunities for improving diagnostics and treatment strategies.

One emerging approach applies ML algorithms to MALDI-TOF MS spectral data. Whereas MALDI-TOF algorithms focus only on the most prominent peaks, ML can uncover subtle patterns, including non-linear relationships and interactions among features, in high-dimensional data [5]. Although several studies have demonstrated the potential of ML-assisted MALDI-TOF MS analysis, the field remains relatively young, and important methodological challenges remain [5]. Studies to date have primarily focused on genera such as *Staphylococcus*, *Streptococcus*, *Escherichia*, *Bacillus*, and *Klebsiella* [5].

Recent studies have demonstrated that combining MALDI-TOF MS with machine learning approaches enables rapid prediction of antimicrobial resistance directly from spectral data [9,10]. Various algorithms, including Random Forest (RF), Support Vector Machine (SVM), Logistic Regression (LR), Gradient Boosting (GB), and neural network-based approaches, have shown promising performance across different bacterial species and antibiotic classes. For example, Weis et al. reported AUROC values of approximately 0.70–0.80 for selected species–antibiotic combinations such as ceftriaxone resistance in *E. coli* and *K. pneumoniae*, and oxacillin resistance in *S. aureus*, while Nguyen et al. demonstrated strong predictive performance for several resistance phenotypes for fluoroquinolones and aminoglycosides (AUROC 0.81–0.84) in *P. aeruginosa* [9,10,11]. Despite these encouraging results, important challenges remain, including limited transparency of preprocessing workflows, restricted availability of datasets and source code, insufficient external validation, and a lack of studies that systematically evaluate the impact of culture conditions, sample storage, and spectral preprocessing on model performance [5]. Collectively, these limitations hinder reproducibility and complicate the implementation of ML-based MALDI-TOF resistance prediction across laboratories.

Additionally, bacterial growth conditions influence MALDI-TOF mass spectra by altering cellular protein expression profiles, resulting in measurable differences in spectral peak patterns and intensities that can influence downstream machine learning analyses. Consequently, culture protocols and sample preparation procedures represent important sources of experimental variability that may impact the reproducibility and transferability of machine learning models across laboratories. Despite this, the influence of culture conditions and other preanalytical factors on ML-based antimicrobial resistance prediction remains insufficiently studied. Therefore, standardized experimental workflows and transparent methodological reporting are essential for future clinical implementation and independent validation of MALDI-TOF-based ML models.

In this study, we aimed to establish a reproducible, end-to-end machine learning pipeline for predicting antibiotic resistance from MALDI-TOF MS spectra, with emphasis on (1) transparent and standardized spectral preprocessing, (2) rigorous model validation through nested cross-validation and multi-model comparison, and (3) systematic evaluation of how culture conditions, extract storage, and spectral alignment affect model performance.

The main contributions of this study are: (i) an open-source MALDI-TOF preprocessing pipeline (MTPP); (ii) systematic comparison of four ML algorithms using statistical testing; (iii) evaluation of extract storage effects on spectral quality and model performance; (iv) isolate-level cross-validation to prevent data leakage; and (v) complete code and data availability to support reproducibility.

## 2. Materials and Methods

### 2.1. Bacterial Isolates

A selection of 282 *E. coli* isolates from two laboratories was used in this study. The collection included 48 *E. coli* isolates from the culture collection of the Laboratory Bacteriology Research (LBR; Department of Diagnostic Sciences, Faculty of Medicine and Health Sciences, Ghent University, Ghent, Belgium), hereinafter referred to as the ‘LBR collection’. The laboratory and clinical isolates of the LBR collection were selected to maximize *E. coli* diversity in terms of clinical conditions and anatomical sites while also including closely related isolates, such as longitudinal samples from the same patient and isolates linked to outbreaks. The LBR collection was used to investigate reproducibility and optimize spectra acquisition. After optimization, the collection was expanded with 234 clinical *E. coli* blood isolates from the culture collection of the Department of Microbiology and Infection Control of the Brussels University Hospital, Brussels, Belgium (UZ Brussel), hereinafter referred to as the ‘UZB collection’ [12].

The identification of the isolates was confirmed by means of MALDI-TOF MS, as previously described [13].

### 2.2. Culture Conditions and Optimization

All 282 *E. coli* isolates were cultured overnight from freezer stock (stored at −80 °C) on MacConkey II agar plates (Becton Dickinson, Erembodegem, Belgium) under aerobic conditions at 37 °C. Subcultures were prepared by using a 1 µL inoculation loop to take a few colonies from each isolate. These were then incubated under the same conditions for 18 h before a protein extraction procedure (see below).

Culture medium comparison and incubation time optimization experiments are provided in Table S4 and Figure S2.

### 2.3. Antibiotic Susceptibility Testing

Antibiotic susceptibility testing was performed by assessing the minimum inhibitory concentration (MIC) (Sensititre™ Custom Plates, Thermo Fisher Scientific, Waltham, MA, USA), as previously described [8,12]. The MIC was assessed for amoxicillin (AMX; 1–16 µg/mL), aztreonam (ATM; 0.5–32 µg/mL), cefepime (FEP; 0.5–16 µg/mL), ceftazidime (CAZ; 0.5–16 µg/mL), colistin (CST; 1–16 µg/mL), meropenem (MEM; 1–16 µg/mL), amoxicillin–clavulanic acid (AMC; 1/2–32/2 µg/mL), piperacillin/tazobactam (TZP; 2/4–32/4 µg/mL), amikacin (AKN; 4–32 µg/mL), tobramycin (TOB; 1–16 µg/mL), tigecycline (TGC; 0.25–8 µg/mL), trimethoprim–sulfamethoxazole (SXT; 1/19–16/304 µg/mL) and ciprofloxacin (CIP; 0.06–8 µg/mL) by using the ISO 20776-1 standard broth microdilution method, as recommended by The European Committee on Antimicrobial Susceptibility Testing (EUCAST) [14]. We used Sensititre™ Custom Plates according to EUCAST guidelines. We used the EUCAST 2024 clinical breakpoints to classify isolates as susceptible, intermediate (=susceptible at increased exposure), and resistant (https://www.eucast.org/clinical_breakpoints, accessed on 16 August 2024).

### 2.4. MALDI-TOF MS Spectra Generation

Protein extracts were prepared by collecting a few colonies from the subculture plates using a 10 µL inoculation loop and resuspending in a 1.5 mL Eppendorf tube containing 300 µL water (Sigma-Aldrich, Merck Life Science BV, Hoeilaart, Belgium). The tubes were briefly vortexed before and after the addition of 900 µL 96% ethanol (VWR, Leuven, Belgium). The suspensions were subsequently centrifuged for 2 min at 11,300 *g*, and the supernatant was removed by decantation of the tube. Residual supernatant was removed by careful pipetting, and the pellet was air-dried for approximately 30 min. After drying the pellet, 80 µL of 70% formic acid (Sigma-Aldrich) and 80 µL of acetonitrile (Sigma-Aldrich) were sequentially added to each tube, and these tubes were centrifuged for 2 min at 11,300 *g*. The supernatant was transferred to a new, sterile 1.5 mL Eppendorf tube and stored at −20 °C.

PMFs were acquired using a MALDI Biotyper^®^ Sirius System (Bruker Daltonics, Bremen, Germany). A total of 1 µL of each protein extract of each isolate was spotted 8 times (technical replicates) within each column on an MSP 96 target polished steel BC (Bruker Daltonics). Each spot was subsequently covered with well-vortexed 1 µL α-Cyano-4-hydroxycinnamic acid (HCCA) matrix (Bruker Daltonics) and was air-dried before placing the target plate in the Sirius instrument.

Subsequently, spectra were generated using FlexControl version 3.4 (build 206.67) software (Bruker Daltonics) and a default-adjusted user method for acquiring spectra in the mass range of 2–15 kDa, with peak selection set to use masses in this range. This *m*/*z* range was selected based on the results of the optimization procedure. The AutoXecute settings were adjusted to save the sum of rejected spectra, which was required to circumvent spectral zerolines (i.e., when no intensities were saved for the *m*/*z* range). The AutoXecute method accumulation settings were set to sum up 240 satisfactory shots in 40-shot steps, with 80 satisfactory shots per raster spot. The initial laser power was set at 30% with a maximal laser power of up to 40%. The generated raw MALDI-TOF spectra were exported as .txt files containing two columns: the *m*/*z* values and the signal intensities.

### 2.5. Characterization of the Data

#### 2.5.1. MALDI-TOF MS Datasets

To evaluate the robustness, reproducibility, and temporal stability of MALDI-TOF MS spectra for *E. coli* isolates and acquire sufficient data for ML modeling, three complementary datasets were defined, derived from data of the entire collection of isolates (LBR+UZB) (Table 1). This strategy was implemented to simulate and test realistic laboratory scenarios where clinical microbiology laboratories may acquire PMFs across different sites, at varying timepoints, or from protein extracts subjected to prolonged storage.

The first dataset (hereinafter referred to as ‘T_1_’) was generated by culturing the LBR+UZB collection from freezer stocks, preparing protein extracts, and acquiring PMFs on a MALDI-TOF MS Sirius system in January 2022 at the clinical microbiology department of the University Hospital of Brussels. The second dataset (hereinafter referred to as ‘T_2_’) was obtained by reanalyzing the same protein extracts used for T_1_ after one year of storage at −20 °C, with PMFs acquired on the MALDI-TOF MS Sirius system at the clinical microbiology department of Ghent University Hospital. The third dataset (hereinafter referred to as ‘T_3_’) was created by reculturing the LBR+UZB collection from freezer stocks, preparing new protein extracts, and acquiring PMFs on the same instrument at Ghent University Hospital in January 2023.

Technical replicates were defined as multiple independent MALDI-TOF measurements of the same protein extract from a given isolate, acquired on the same or on different days. T_1_ and T_2_ represent repeat measurements of the same initial sample preparation, with T_2_ performed approximately one year after T_1_ to assess long-term technical reproducibility. In contrast, T_3_ consists of biological replicates obtained by reculturing the original isolates, introducing additional biological variability beyond that of technical replicates.

#### 2.5.2. Metadata: Antibiotic Resistance Profiles

Metadata included isolate identifiers and antibiotic susceptibility profiles for 12 antibiotics based on MIC testing (classified as R for resistant or S for susceptible). All metadata were merged into a single dataset (.csv file) and linked to the corresponding MALDI-TOF entries via unique isolate IDs.

### 2.6. Construction of a MALDI-TOF MS Data Processing Pipeline (MTPP)

The raw PMFs were processed using a custom MALDI-TOF MS data processing pipeline (MTPP) built in R with the MALDIquant package (v1.22.3) [15], supplemented by in-house scripts. The pipeline comprised eight sequential steps: (1) intensity variance stabilization (sqrt method) [16]; (2) smoothing (Savitzky–Golay filter, halfWindowSize = 10) [17]; (3) baseline correction (SNIP, iterations = 100) [18,19]; (4) normalization (total ion current, TIC) [20,21,22]; (5) spectral alignment (LOWESS-based warping, halfWindowSize = 20, reference peak SNR ≥ 2, tolerance = 0.2 *m*/*z*) [15]; (6) peak detection (MAD noise estimation, halfWindowSize = 20, SNR ≥ 3) [23]; (7) peak binning (tolerance = 0.002 *m*/*z*) and filtering (minimum peak frequency ≥ 10% of spectra); and (8) feature matrix construction with zero-threshold imputation (intensity values < 1 × 10^−6^ set to zero to disambiguate absent peaks from interpolation artefacts). The SNR threshold for peak detection was increased from the commonly used value of 2 to 3 to reduce false positive peak detections that could confuse downstream classifiers. The minimum peak frequency was raised from 5% to 10% to remove sporadic noise peaks while retaining biologically relevant features. Two parallel feature matrices were generated—one from aligned spectra (ALI) and one from non-aligned spectra (NALI)—to enable comparative assessment of the alignment effect. All parameter settings and justifications are listed in Table S1.

The final aligned feature matrix (T_1+3_) comprised 4468 spectra × 121 *m*/*z* features (mass range 2084–12,977 Da); the non-aligned matrix comprised 4468 spectra × 132 features. Each row represents one PMF linked to its corresponding isolate, and each column represents a binned *m*/*z* peak with interpolated intensity values.

### 2.7. Development of ML Models to Predict Antibiotic Resistance

For the predictive classification of antibiotic resistance from MALDI-TOF data, four machine learning algorithms were evaluated using the scikit-learn Python library (v1.6.1): Random Forest (RF), Logistic Regression (LR), Support Vector Machine (SVM), and Gradient Boosting (GB). RF was selected as the primary model based on its established performance with high-dimensional spectral data [9], native handling of non-linear feature interactions, and interpretability through feature importance analysis. The remaining three algorithms served as comparators to validate the choice of RF.

#### 2.7.1. Data Preprocessing and Splitting

The aligned MALDI-TOF feature matrix (T_1+3_) and antibiotic susceptibility metadata were merged via shared isolate identifiers. Antibiotic susceptibility profiles were binarized by grouping susceptible (S) and intermediate (I, susceptible at increased exposure) phenotypes as the negative class (0) and resistant (R) phenotypes as the positive class (1). Meropenem (no resistant isolates) and colistin (only 3 resistant isolates, insufficient for cross-validated evaluation) were excluded, yielding 11 antibiotics for modeling.

Model evaluation employed a nested cross-validation framework to prevent data leakage and hyperparameter overfitting. The outer evaluation used GroupShuffleSplit (n_splits = 5, test_size = 0.2), where groups were defined at the isolate level (279 unique isolates), ensuring that all spectra (~16 per isolate across T_1_ and T_3_) from the same isolate remained in either the training or testing set. Within each outer fold, feature selection and hyperparameter tuning were performed using an inner GroupKFold (n_splits = 5) cross-validation. As supplementary validation, StratifiedGroupKFold (n_splits = 5) was also performed independently to verify the stability of estimates across alternative splitting strategies.

#### 2.7.2. Feature Selection and Model Training

Recursive Feature Elimination with Cross-Validation (RFECV) was applied independently within each outer training fold to identify the optimal feature subset for each antibiotic. At each iteration, 10% of the remaining features were removed (step = 0.1). The estimator used for ranking was an RF classifier with hyperparameters determined by nested tuning (see below). The scoring metric was the F1-score for binary tasks. Internal cross-validation used GroupKFold (n_splits = 5), respecting isolate-level grouping. The number of selected features ranged from 1 (amikacin, cefepime, piperacillin–tazobactam, tigecycline, tobramycin) to 85 (aztreonam), with ciprofloxacin requiring 13 features.

Hyperparameter optimization was performed via nested RandomizedSearchCV (n_iter = 50, inner CV: GroupKFold, n_splits = 5) within each outer training fold. For the primary RF model, the search space comprised: n_estimators ∈ {100, 300, 500}, max_depth ∈ {None, 20, 30}, min_samples_leaf ∈ {1, 3, 5}, max_features ∈ {‘sqrt’, ‘log2’, 0.3}, and class_weight ∈ {‘balanced’, ‘balanced_subsample’}. Comparable search spaces were defined for the three comparator models (Table S5). Representative optimized Random Forest hyperparameter configurations and the corresponding numbers of RFECV-selected features for each antibiotic are provided in Table S6. After feature selection and tuning, the final model was trained on the full outer training set and evaluated on the held-out outer test fold. All reported performance metrics were derived from these outer test folds to ensure unbiased estimation.

#### 2.7.3. Class Imbalance Handling

Class imbalance was addressed through three complementary strategies: (1) cost-sensitive learning via class_weight = ‘balanced_subsample’ in RF, which adjusts class weights inversely proportional to class frequencies within each bootstrap sample; (2) threshold optimization, where the binary classification threshold was set to maximize the F1-score on the precision–recall curve of the training set rather than using the default value of 0.5; and (3) F1-based scoring during both RFECV feature selection and hyperparameter tuning, ensuring that model selection prioritized minority class detection over overall accuracy.

#### 2.7.4. Model Evaluation

Model performance was assessed using multiple classification metrics to ensure robustness across imbalanced phenotype classes. For each prediction task, we generated confusion matrices, saved in both tabular and heatmap formats, to provide a detailed overview of classification outcomes.

We reported the Area Under the Receiver Operating Characteristic Curve (AUROC) and the area under the precision–recall curve (AUPRC) as primary metrics. These are particularly suited for tasks with class imbalance, i.e., when one class (e.g., resistant isolates) is substantially underrepresented or overrepresented compared to the other class (e.g., susceptible isolates), typically defined as a class ratio below 20% or above 80%. Such an imbalance is common in antibiotic resistance phenotyping, where, for certain antibiotics, resistant isolates may constitute only a small fraction of the tested population. AUROC—computed as the area under the ROC curve, which plots the True Positive Rate (TPR) against the false positive rate (FPR) at various classification thresholds—summarizes the model’s ability to distinguish between classes. AUPRC—computed as the area under the precision–recall curve, which plots precision against recall at different classification thresholds—emphasizes precision and recall trade-offs for the positive (resistant) class, offering a more informative evaluation when the positive class is rare.

AUROC values were interpreted as: poor (<0.70), fair (0.70–0.79), considerable (0.80–0.89), and excellent (≥0.90). Scores ≤ 0.60 were considered equivalent to random chance [24].

To complement these, we included accuracy, precision, recall, F1-score, Matthews Correlation Coefficient (MCC), and Cohen’s Kappa (Table 2). MCC was included because it provides a balanced measure even under severe class imbalance, as it accounts for all four confusion matrix categories. The definitions of all metrics are provided in Table 2.

For binary classification, antibiotic susceptibility profiles were binarized by grouping intermediate (I, susceptible at increased exposure) and susceptible (S) phenotypes as the negative class and resistant (R) phenotypes as the positive class. This binarization reflects the clinical relevance of identifying resistance.

All evaluation metrics were exported as .csv files for downstream analysis.

#### 2.7.5. Multi-Model Comparison and Statistical Analysis

To validate the choice of RF, LR, SVM, and GB, each was trained on the identical RFECV-selected feature sets and evaluated on the same outer test folds. Pairwise model comparisons were tested via McNemar’s chi-squared test, yielding six comparisons per antibiotic. Bootstrap 95% confidence intervals (1000 replicates, percentile method) were computed for accuracy, F1-score, precision, recall, MCC, and AUROC. Additionally, permutation importance analysis (10 repeats, F1 scoring) was performed to identify the most discriminatory *m*/*z* features for each antibiotic. Learning curves and calibration curves (reliability diagrams) were generated to assess overfitting and probability calibration, respectively.

### 2.8. Optimizations for PMF Generation and Data Acquisition

Prior to investigating the LBR+UZB collection, the LBR collection was used to evaluate and refine culture conditions and MALDI-TOF MS data acquisition parameters. Various settings were compared to assess their impact on spectral quality and consistency, with selection based on practicality, the number of detectable peaks (as a proxy for information content), and alignment with MLST results. For example, conditions such as the use of blood agar plates versus MacConkey agar were evaluated, with preference given to those offering better correspondence with MLST clustering (Figure S2) or higher spectral resolution.

These optimizations focused on (1) the effect of TSA+5% sheep blood culture medium vs. MacConkey medium (selective culture medium for Gram-negative bacteria) on the generated PMFs, (2) reproducibility through similarity analyses between MALDI-TOF MS analyses of protein extracts derived from isolates cultured on MacConkey medium between three measurements (baseline measurement, baseline + 2 months, baseline + 4 months), (3) the culture incubation time and its effect on the peaks in the generated PMFs, (4) the peptide detection range (*m*/*z* range), and (5) using the direct smear method (i.e., directly transferring colony material from the culture plate to the MALDI-TOF target plate) versus the use of protein extracts.

The raw PMF data from the optimization experiments were analyzed using the BioNumerics version 8.1.1 platform (BioMérieux, Schaerbeek, Belgium). The PMFs were preprocessed by performing baseline subtraction, noise computation, and smoothing. The peak detection parameter of peak filtering was set with an SNR of five. Cluster analyses were generated with similarity coefficients based on the curve and Pearson correlation. The ‘UPGMA’ cluster analysis method was selected. Similarity matrices were generated by performing hierarchical clustering analysis on the MALDI-TOF data and annotated with the MIC and MLST data.

### 2.9. Data Analysis

#### 2.9.1. Principal Component Analysis of the MALDI-TOF Feature Matrix

Principal component analysis (PCA) was conducted to identify principal components explaining the most variance in the MALDI-TOF MS data. The analysis utilized the ‘PCA’ class from the ‘sklearn.decomposition’ module of the ‘scikit-learn’ package in Python. The input data consisted of the preprocessed MALDI-TOF feature matrix derived from datasets T_1_, T_2_, and T_3_. Additionally, PCA was performed on the RFECV-selected feature subsets for each antibiotic, using both two-component and automatic component selection (95% cumulative variance threshold), to visualize class separability in reduced dimensionality.

#### 2.9.2. Reproducibility and Alignment Effect on PMF Similarity Across Technical Replicates

Similarity analyses were performed to assess: (1) the reproducibility of the MALDI-TOF MS technique by comparing technical replicate PMFs and (2) the effect of the MTPP alignment procedure on PMF similarity.

Similarity percentages were computed between replicate PMFs for both aligned and non-aligned spectra using the SpectrumSimilarity function from the ‘OrgMassSpecR’ package in R (https://github.com/OrgMassSpec/OrgMassSpecR, accessed on 29 June 2026). Alignment was included to assess whether preprocessing via peak alignment (i.e., correcting for minor *m*/*z* shifts across spectra) improves the comparability of replicate PMFs, particularly across timepoints and biological replicates. This resulted in two similarity matrices—one for aligned PMFs and one for non-aligned PMFs—which were exported as .csv files for further analysis. An in-house Python script was then used to extract and compare the similarity values across three analytical levels:

Within-timepoint reproducibility: Similarity values were calculated for all pairwise combinations of replicate PMFs within each dataset (T_1_, T_2_, and T_3_).

Between-timepoint reproducibility: For each isolate, pairwise similarity scores were computed between technical replicates of T_1_ and T_2_ (same sample preparation, different acquisition times), as well as between T_1_ and T_3_ and between T_2_ and T_3_ (introducing biological variability due to reculturing in T_3_).

Combined dataset configurations: Comparisons across merged datasets (T_1+2_, T_1+3_, T_2+3_) were used to evaluate reproducibility and variability over time, incorporating both technical and biological effects.

Violin box plots visualizing the distributions of similarity percentages were generated in Python. Statistical significance between aligned and non-aligned similarity scores was assessed using the Wilcoxon signed-rank test, with significance defined as *p* < 0.001.

## 3. Results

The influence of culture conditions and standardization on PMF profiles and reproducibility is described below.

The evaluation of the influence of different parameters and standardization on PMF generation is presented in Table S4. Briefly, further analyses were performed with cultures derived from MacConkey medium—incubated for 18 h—which presented better reproducibility and specificity than TSA + 5% sheep blood medium-derived cultures. A PMF mass range of 2–15 kDa was used as the *m*/*z* default range.

### 3.1. Isolate Characterization

**Figure 1:**
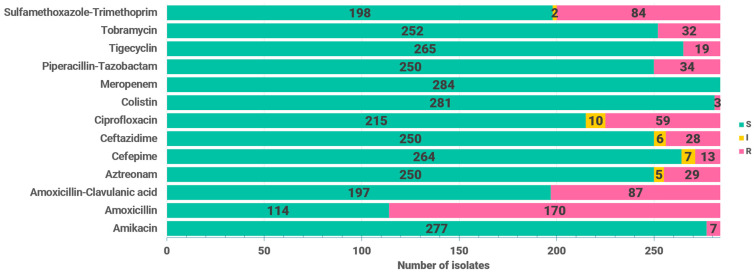
Summary of antibiotic susceptibility testing phenotypes according to minimum inhibitory concentration (MIC) for 284 clinical isolates. Number of isolates with a susceptible phenotype (green), susceptible at increased exposure (orange) and resistant phenotype (red) of each antibiotic.

A summary of the antibiotic susceptibility phenotypes of the isolates, as determined by MIC assays for 13 antibiotics, is presented in Figure 1.

For nine out of 13 antibiotics, including amikacin, aztreonam, cefepime, ceftazidime, colistin, meropenem, piperacillin–tazobactam, tigecycline, and tobramycin, a high class imbalance in resistance phenotypes was observed (a positive/resistant class ratio <20% or >80%). Generally, the phenotypically susceptible isolates (range 88.03–100%) made up the vast majority compared to phenotypically resistant isolates (range 0–11.97%). No phenotypically resistant isolates against meropenem were observed; meropenem was therefore excluded from subsequent analyses. Colistin (3/284 resistant, 1.1%) was retained in the similarity and preprocessing analyses but excluded from ML modeling due to insufficient resistant isolates for reliable cross-validated evaluation. For four out of 13 antibiotics, including amoxicillin, amoxicillin–clavulanic acid, ciprofloxacin, and sulfamethoxazole–trimethoprim, both phenotypic classes had >20% of occurrences.

### 3.2. MALDI-TOF Preprocessing Pipeline (MTPP)

#### 3.2.1. Effect of Alignment on the Similarity Percentages Between Replicate PMFs

Initial clustering analyses revealed that replicate PMFs from different subsets (e.g., timepoints or conditions) did not cluster together as expected, suggesting underlying inconsistencies in the PMFs. Upon closer visual inspection of raw and preprocessed spectra, we identified systematic peak shifts between technical and biological replicate PMFs. These spectral shifts likely contributed to the unexpectedly low similarity percentages observed between technical and biological replicates. To address this issue, we implemented a comparative analysis of replicate PMF similarity with and without alignment correction (referred to as ‘ALI’ and ‘NALI’, respectively). Alignment served as a shift correction and normalization approach to improve intra-isolate spectral reproducibility and to standardize peak positions across PMFs prior to downstream analysis.

**Figure 2:**
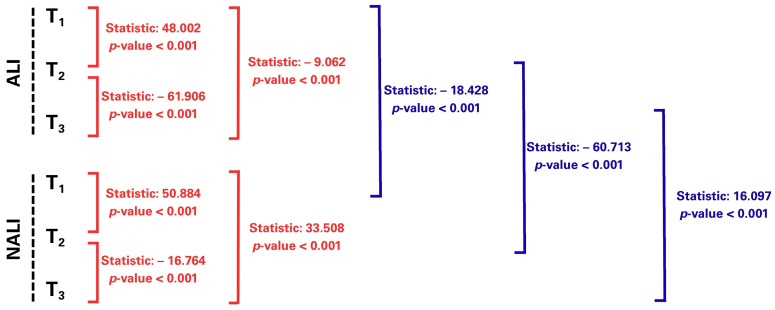
The Wilcoxon rank-sum test statistics for pairwise comparisons of replicate MALDI-TOF MS spectral similarity distributions. **Red brackets** denote comparisons between datasets processed using the same preprocessing approach (within ALI or within NALI), whereas **blue brackets** denote comparisons between the corresponding aligned (ALI) and non-aligned (NALI) datasets at each time point (T_1_–T_3_). The Wilcoxon rank-sum test statistic and associated *p*-value are shown for each comparison.

The Wilcoxon rank-sum statistics between the ALI and NALI similarity percentages are shown in Figure 2. All dataset comparisons showed statistically significant differences (*p*-value < 0.001), indicating that the data have different distributions. The positive statistics (s) for the comparisons T_1_↔T_2_ in the ALI data and T_1_↔T_2_ and T_1_↔T_3_ in the NALI data indicate that the first dataset tends to have higher similarity values. In contrast, negative statistics, such as the comparisons T_2_↔T_3_ and T_1_↔T_3_ in the ALI data and T_2_↔T_3_ in the NALI data, indicate that the second dataset tends to have higher similarity values. Comparative analysis of each dataset with and without alignment showed that the ALI dataset has significantly higher values than the NALI dataset for T_3_, whereas the NALI dataset has significantly higher values than the ALI dataset for T_1_ and T_2_.

**Figure 3:**
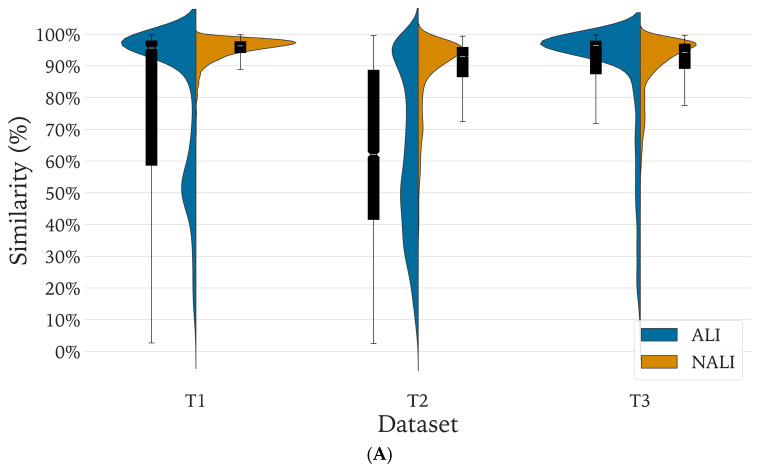

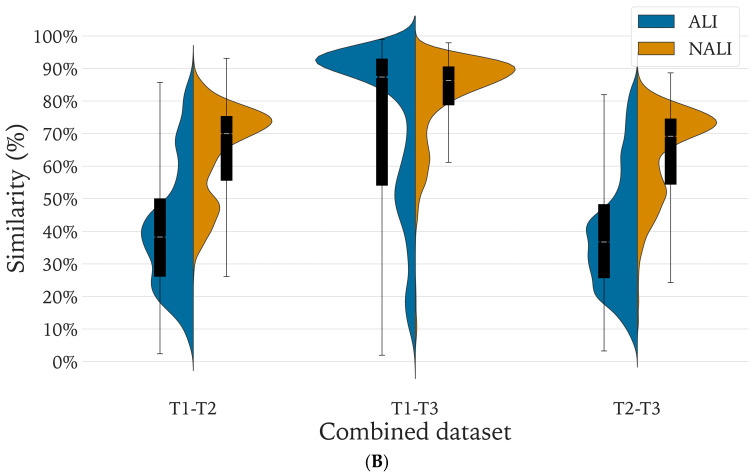
Violin box plots of the similarity percentages between replicate PMFs within each dataset and with or without alignment (**panel A**). Violin box plots of the similarity percentages between replicate PMFs within different dataset combinations and with or without alignment (**panel B**). ALI, data was aligned; NALI, data was not aligned.

The similarity comparison between replicate PMFs at T_1_, T_2_, and T_3_ with and without the alignment procedure during MTPP preprocessing is shown as a split violin plot in Figure 3A. The similarity percentages between aligned replicate PMFs in T_1_ were in the range of 2.64–99.76%, with a median of 95.64%. In T_2_, the similarity percentages for the aligned PMFs ranged between 2.47 and 99.56%, with a median of 62.06%. For T_3_, the range was 4.17–99.78%, with a median of 96.47%. The similarity percentages between non-aligned replicate PMFs in T_1_ were in the range of 56.74–99.77%, with a median of 96.31%. In T_2_, the similarity percentages for the non-aligned PMFs ranged between 29.05 and 99.37%, with a median of 92.89%. For T_3_, the range was 46.29–99.67%, with a median of 94.23%.

The comparison between replicate PMFs for different combinations of datasets T_1_, T_2_, and T_3_ and with and without the alignment procedure (ALI versus NALI) during MTPP-preprocessing is shown as a split violin plot in Figure 3B. The similarity percentage comparison of aligned PMFs from T_1+3_ was in the range of 1.93–98.96%, with a median of 87.37%, whereas the similarities for T_1+2_ and T_2+3_ were very similar, with ranges of 2.35–91.93% and 3.24–90.10% and medians of 38.25% and 36.73%, respectively. For the non-aligned PMFs, T_1+3_ was in the range of 5.19–97.88%, with a median of 86.31%, whereas the similarities for T_1+2_ and T_2+3_ were very similar, with ranges of 24.22–93.10% and 10.47–88.63% and medians of 69.92% and 69.18%, respectively.

In summary, these results unexpectedly indicated superior similarity percentages between technical and biological replicate PMFs when alignment was not performed. T_1+3_ presented the highest similarity percentages between the technical and biological replicate PMFs, whereas upon introduction of PMFs from T_2_, more variability and thus lower similarity percentages between technical and biological replicate PMFs were observed. Due to these observations, we examined if modifying the ‘t’ parameter from the SpectrumSimilarity-function caused the difference between alignment and non-alignment (Table S2). The ‘t’ parameter represents a numeric value specifying the tolerance used to align the *m*/*z* values of two PMFs when comparing the PMFs and to calculate a similarity percentage. If ‘t’ ≠ 0, then alignment is performed once as part of the MTPP during PMF preprocessing and a second time by the SpectrumSimilarity function, possibly causing excessive alignment. Therefore, we changed the hyperparameter value of ‘t’ from 3 to 0 and did the same investigation. Additionally, due to differences in the freshness of the samples used for the generation of the PMFs (T_1_, T_3_ (fresh preparations) versus T_2_ (freeze-stored samples)) and the discrepancies observed in the similarity percentage comparisons above, we also examined if exclusion of T_2_ improved the similarity percentages between technical and biological replicate PMFs. Therefore, we processed the data from T_1_ and T_3_ through the MTPP and compared the similarity percentages again with and without alignment. The descriptive statistics for these analyses are shown in Table S2.

The results indicated that alignment significantly impacted the similarity percentages between technical replicate PMFs of T_2_ in T_1+2+3_ with ‘t’= 3 but not the similarity percentages between technical replicate PMFs of T_1_ and T_3_. The median similarity percentages for technical replicate PMFs were 95.64% (ALI) and 96.31% (NALI) for T_1_, 62.06% (ALI) and 92.89% (NALI) for T_2_, and 96.47% (ALI) and 94.23% (NALI) for T_3_. The results for T_1+2+3_ with ‘t’= 0 presented similar results, where a significant increase in similarity percentages was observed for T_2_, whereas T_1_ and T_3_ did not show significantly different results. Here, the median similarity percentages for technical replicate PMFs were 95.64% (ALI) and 96.28% (NALI) for T_1_, 62.06% (ALI) and 92.06% (NALI) for T_2_, and 96.47% (ALI) and 93.63% (NALI) for T_3_. In the combined dataset T_1_ + T_3_ with ‘t’= 3, the median similarity percentages were 97.70% (ALI) and 94.59% (NALI) for T_1_ and 97.15% (ALI) and 94.88% (NALI) for T_3_. Comparison of the similarity percentages of replicate PMFs in T_1_–T_3_ showed medians of 92.15% (ALI) and 85.12% (NALI). Detailed descriptive statistics are shown in Table S2.

In summary, we found that changing the parameter ‘t’ did not significantly affect the results (<1% difference between a ‘t’ of 0 or 3) for T_1+2+3_. The similarity percentages from T_2_ were significantly affected by performing alignment, with superior similarities observed between replicate PMFs without alignment and with alignment. This was not the case for T_1_ and T_3_, both showing similar percentages with and without alignment. On the other hand, for T_1+3_, alignment did improve the similarity percentages between replicate PMFs. In light of these findings, we chose to continue with the data from T_1_ and T_3_ to train the ML models, thereby reducing spectral interference/variability introduced by storage-degraded T_2_ spectra.

#### 3.2.2. Characterization of the MALDI-TOF Feature Matrix

The feature matrices generated by the MTPP contain rows representing individual PMFs and columns representing binned *m*/*z* peak features. For T_1+2+3_, the feature matrix dimensions were 6692 × 319 (ALI) and 6692 × 292 (NALI), with *m*/*z* ranges of 2083.9–12,978.0 Da and 2085.2–12,973.3 Da, respectively. For the primary dataset T_1+3_, the dimensions were 4468 × 121 (ALI) and 4468 × 132 (NALI), with *m*/*z* ranges of 2083.7–12,976.8 Da and 2082.5–12,973.3 Da, respectively. The reduction from 216 to 121 features (ALI) compared with our preliminary analysis reflects the updated MTPP parameters: a stricter peak detection threshold (SNR ≥ 3 vs. 2) and an increased minimum peak frequency filter (10% vs. 5%) removed sporadic noise peaks while retaining biologically relevant features.

**Figure 4:**
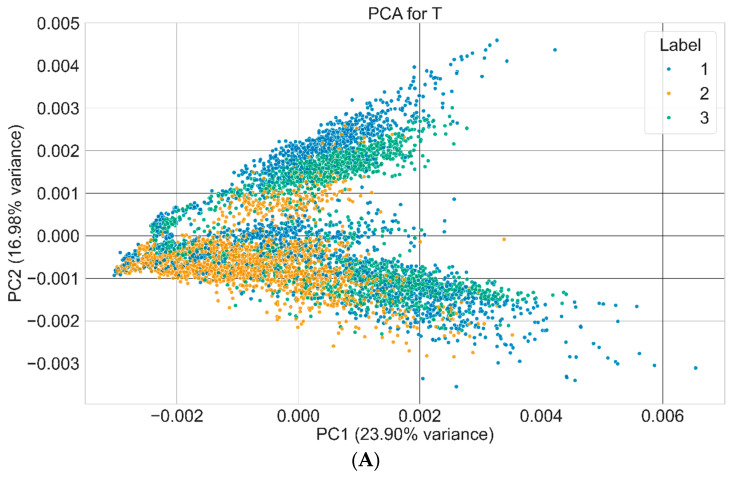

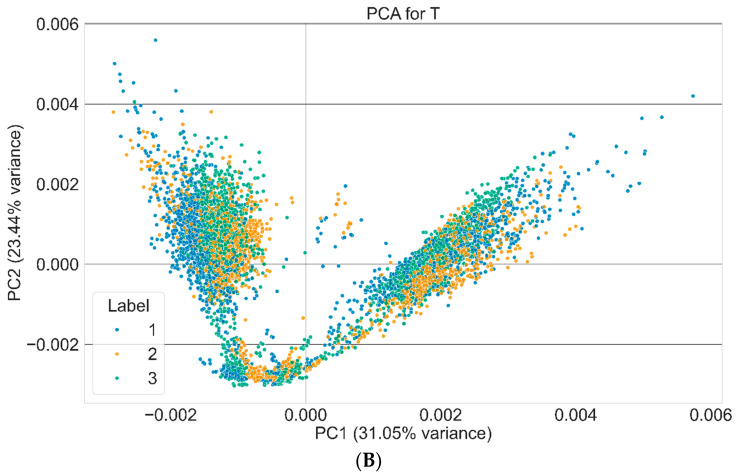
Principal component analysis (PCA) of the MALDI-TOF MS peak feature matrices of datasets T_1_, T_2_, and T_3_ acquired through a MALDI-TOF MS data preprocessing pipeline with alignment (**Panel A**) and without alignment (**Panel B**). The spectral data is colored based on the dataset (T_1_, T_2_, and T_3_).

To uncover underlying structures related to the specific datasets and the alignment procedure, we conducted a principal component analysis (PCA) on the MALDI-TOF feature matrix acquired as output from the MTPP. The PCA biplots for the MALDI-TOF feature matrices with alignment processing and without alignment are shown in Figure 4A and Figure 4B, respectively. The PCA biplots show the distribution of the samples along the first two principal components (PC1 and PC2), which together explain a substantial proportion of the variance in the data. The data is color-coded based on dataset alias (T_1_, T_2_, and T_3_).

The PCA biplot in Figure 4A, based on aligned PMFs, shows that PC1 and PC2 account for 23.90% and 16.98% of the total variance, respectively, together explaining 40.88% of the overall variance. While PC1 and PC2 provide partial separation between the datasets, the observed clustering is not fully distinct. T_2_ tends to group toward the left-central region of the plot, whereas T_1_ and T_3_ are more broadly dispersed across the central, upper, and right-hand regions. T_1_ displays the widest spread and occupies the middle of the plot, while T_3_ overlaps considerably with T_1_. The presence of overlap among the datasets—particularly between T_1_ and T_3_—suggests shared spectral characteristics, potentially due to biological similarities (as both originate from the same isolates, albeit T_3_ was recultured). Thus, while some degree of clustering is visible, it should be interpreted with caution and does not indicate complete dataset separation.

The PCA biplot derived from PMFs without alignment is shown in Figure 4B. PC1 accounts for 31.05% of the variance, while PC2 explains 23.44%, cumulatively capturing 54.49% of the overall variance. Interestingly, no clear distinctions between the three datasets can be observed when alignment was not performed on the PMFs in the MTPP.

### 3.3. Machine Learning Classification Model Performance

#### 3.3.1. Primary Model Performance for Antibiotic Resistance Prediction

**Figure 5:**
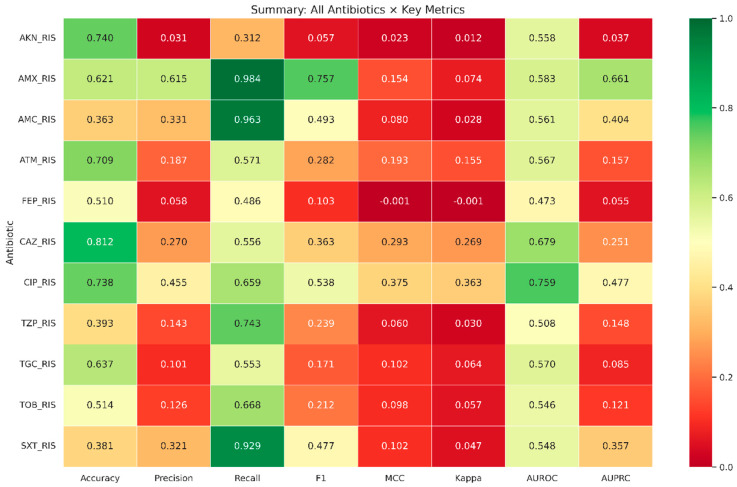
Summary heatmap of primary Random Forest classifier performance across 11 antibiotics. Each cell displays the mean metric value across five outer GroupShuffleSplit folds. Metrics shown: AUROC, AUPRC, accuracy, precision, recall, F1-score, MCC, and Cohen’s Kappa. Color intensity scales from low (dark) to high (bright) performance. Antibiotics are ordered alphabetically. Ciprofloxacin and ceftazidime show the strongest overall performance; amikacin, cefepime, and piperacillin–tazobactam show near-chance classification across all metrics.

The performance of the primary RF classifier, evaluated via 5-fold GroupShuffleSplit cross-validation on the aligned T_1+3_ dataset, varied notably across the 11 modeled antibiotics (Table 3 and Table 4, Figure 5). RFECV selected between one and 85 features per antibiotic (Table 5). Performance metrics represent the mean across five outer folds, with 95% bootstrap confidence intervals (1000 replicates).

**Figure 6:**
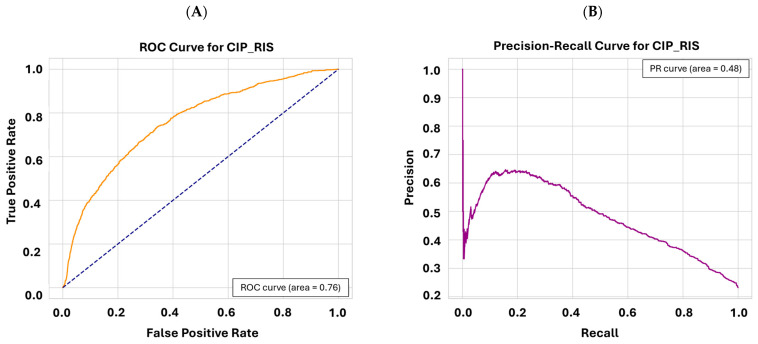
Receiver operating characteristic (ROC) curve (**A**) and precision–recall (PR) curve (**B**) for ciprofloxacin resistance prediction using the primary Random Forest classifier. (**A**) ROC curve showing AUROC = 0.76 [95% CI: 0.74–0.77]. The dashed diagonal line represents the performance of a random classifier (AUROC = 0.50). (**B**) PR curve showing AUPRC = 0.48. Both curves were generated from aggregated predictions of the outer test folds obtained using 5-fold GroupShuffleSplit cross-validation.

The best performance was observed for ciprofloxacin, which achieved an AUROC of 0.759 [95% CI: 0.742–0.775], an F1-score of 0.538, a precision of 0.455, a recall of 0.659, and an MCC of 0.375 using 13 RFECV-selected features. Per-fold F1-scores ranged from 0.415 to 0.677 (mean 0.526, SD 0.094), indicating moderate but consistent discriminative ability across cross-validation splits (Figure 6).

Ceftazidime achieved an AUROC of 0.679 [0.649–0.711] and an F1-score of 0.363, with an MCC of 0.293 using 37 features. Amoxicillin achieved the highest F1-score across all antibiotics (0.757), driven by high recall (0.984) but with modest precision (0.615) and AUROC (0.583 [0.564–0.600]). Sulfamethoxazole–trimethoprim and amoxicillin–clavulanic acid showed F1-scores of 0.477 and 0.493, respectively, but low MCC values (0.102 and 0.080), indicating limited discriminative ability beyond class frequency effects. Aztreonam yielded an AUROC of 0.567 [0.537–0.598] and an F1-score of 0.282 using the largest feature set (85 features).

For three antibiotics—amikacin (class ratio 45.5:1), cefepime (22.3:1), and tigecycline (14.5:1)—no model achieved clinically meaningful performance. AUROC values were 0.558, 0.473, and 0.570, and F1-scores were 0.057, 0.103, and 0.171, respectively. RFECV selected only a single *m*/*z* feature for each, and MCC values were near zero or negative (0.023, −0.001, 0.102), confirming near-random classification. These results are attributable to extreme class imbalance combined with an absence of multi-peak resistance signatures in the 2–15 kDa mass range. Detailed metrics for these antibiotics are presented in Table 4.

Piperacillin–tazobactam (AUROC 0.508, F1 0.239, MCC 0.060) and tobramycin (AUROC 0.546, F1 0.212, MCC 0.098) also showed poor performance, each relying on a single RFECV-selected feature. These antibiotics are reported in the supplementary results alongside amikacin, cefepime, and tigecycline.

#### 3.3.2. Feature Selection

RFECV aggressively reduced the feature space for all antibiotics (Table 5). Five antibiotics (amikacin, cefepime, piperacillin–tazobactam, tigecycline, tobramycin) were reduced to a single *m*/*z* feature, suggesting that no robust multi-peak resistance signature exists for these drugs within the MALDI-TOF mass range. In contrast, aztreonam retained 85 of 121 features (29.8% removed), indicating a more complex spectral signature. Ciprofloxacin, amoxicillin, ceftazidime, and sulfamethoxazole–trimethoprim required 13–37 features, consistent with intermediate signature complexity.

#### 3.3.3. Multi-Model Comparison

**Figure 7:**
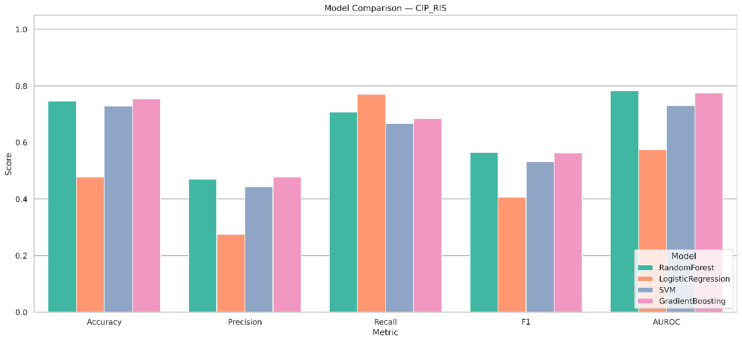
Multi-model comparison of classification performance for ciprofloxacin resistance prediction. Bar chart comparing Random Forest (RF), Logistic Regression (LR), Support Vector Machine (SVM), and Gradient Boosting (GB) across evaluation metrics. All models were trained on the same RFECV-selected feature set (13 features) and evaluated on identical outer test folds. RF and GB performed comparably (McNemar’s *p* = 0.17), while both significantly outperformed LR and SVM (*p* < 0.01).

To validate the choice of RF, three additional classifiers (LR, SVM, GB) were trained on the RFECV-selected features and evaluated on identical outer test folds (Table 6). For ciprofloxacin, RF achieved the highest AUROC (0.782), followed by GB (0.775), SVM (0.730), and LR (0.574). McNemar’s test confirmed that RF significantly outperformed LR (χ^2^ = 724.7, *p* < 0.001) and SVM (χ^2^ = 6.9, *p* = 0.009), while RF and GB did not differ significantly (χ^2^ = 1.8, *p* = 0.17). For ceftazidime, GB achieved the highest AUROC (0.800), followed by RF (0.742), SVM (0.669), and LR (0.624); RF significantly outperformed LR (χ^2^ = 593.0, *p* < 0.001) but did not differ from GB (χ^2^ = 1.2, *p* = 0.28). Across all 11 antibiotics, RF significantly outperformed LR in every case (all *p* < 0.01). RF and GB performed comparably for five of 11 antibiotics (*p* > 0.05). The comparative performance of the four classifiers for ciprofloxacin across all evaluation metrics is summarized in Figure 7. Full pairwise McNemar results are presented in Table S7.

#### 3.3.4. Cross-Validation Stability

The concordance between primary (GroupShuffleSplit) and supplementary (StratifiedGroupKFold) evaluations supported the stability of performance estimates. For ciprofloxacin, the F1-scores were 0.538 (GSS) vs. 0.527 (SGKF); for ceftazidime, 0.363 vs. 0.415; and for amoxicillin, 0.757 vs. 0.765. Bootstrap 95% confidence intervals for the primary evaluation were narrow for well-performing antibiotics (e.g., ciprofloxacin AUROC: 0.742–0.775) and wider for poorly performing ones (e.g., amikacin AUROC: 0.514–0.603), reflecting the instability inherent to extreme class imbalance.

#### 3.3.5. Impact of Dataset Configuration and Alignment on Predictive Performance

To assess the influence of dataset design and preprocessing on model performance, we compared the RF classifier across multiple configurations of aligned (ALI) and non-aligned (NALI) MALDI-TOF MS data from datasets T_1_, T_2_, and T_3_ (Table S3). Each configuration was evaluated using the same cross-validation framework described above.

The following comparison uses the single-split evaluation reported in Table S3, conducted to isolate the effect of dataset configuration while controlling for other variables.

The baseline configuration (T_1+3_—ALI) consistently provided the most balanced performance across metrics (cf. Table 3 and Table 4). This setup demonstrated reliable identification of susceptible isolates (high TN counts) and yielded moderate AUROC values for most antibiotics (e.g., 0.8680 for ciprofloxacin, 0.7141 for ceftazidime). Ciprofloxacin, in particular, showed strong precision (91.00%) and a solid F1-score (51.12%), while sulfamethoxazole–trimethoprim achieved the highest F1-score among moderately performing antibiotics (30.18%).

Adding dataset T_2_ (T_1+2+3_—ALI) introduced greater representation of the different phenotypes and spectral variation, which improved recall for several antibiotics but often at the expense of precision. For instance, recall for sulfamethoxazole–trimethoprim increased from 20.49% to 25.00%, and the F1-score rose from 30.18% to 34.89%, while precision remained stable (57.75%). However, this came with a rise in FPs across several antibiotics. The model continued to struggle to identify resistant isolates for amikacin and cefepime (TP = 0), despite high specificity, resulting in zero recall and precision.

Using non-aligned data (T_1+3_—NALI) generally degraded performance, particularly by increasing FNs, leading to lower recall and F1-scores. For example, recall dropped for tigecycline from 4.17% (NALI) to 2.08% (T_1+2+3_—ALI), and the F1-score remained below 10% across all configurations. Amoxicillin, however, retained relatively stable performance across configurations, with recall hovering around 53–54% and an F1-score above 63%.

Interestingly, colistin predictions improved in recall when using non-aligned data across a broader dataset (T_1+2+3_—NALI), reaching 41.67% recall and an F1-score of 58.82%—a notable increase compared to 18.75% in the baseline. AUPRC also increased from 0.7556 to 0.8715, indicating improved precision–recall balance in this context, despite the usual challenges associated with non-aligned data.

Across all configurations, ceftazidime consistently demonstrated robust performance, with precision above 90% and AUROC values ranging from 0.6752 to 0.7547, suggesting that the model could reliably detect resistance for this antibiotic even under alignment variation.

In summary, the T_1+3_—ALI configuration provided the most consistent, balanced precision and recall across phenotypes. While including T_2_ introduced more variability and improved recall for some targets, it also increased FPs and reduced model stability. Non-aligned data tended to underperform in most cases, confirming the importance of spectral alignment in MALDI-TOF-based prediction pipelines.

## 4. Discussion

### 4.1. Impact of Culture Medium and Incubation Time on PMF Spectral Quality

The culture medium and incubation time significantly influenced spectral variation and the number of peaks detected. Isolates grown on TSA+5% SB plates exhibited greater spectral variation than those grown on MacConkey plates. Moreover, MALDI-TOF MS data generated from MacConkey-derived isolates showed stronger correspondence with MLST-based distinctions than TSA+5% SB (Figure S2). Given MacConkey plates’ ability to provide higher-resolution PMFs within a clinically practical 18 h incubation and with essential peaks within the 2–15 kDa range, we selected these conditions as our standard protocol. This choice has direct implications for clinical translation: laboratories using different culture media would generate systematically different spectra from the same isolates, potentially rendering ML models trained at one site ineffective at another. Standardizing culture conditions is therefore a prerequisite for inter-laboratory model portability—a factor rarely addressed in existing ML-MALDI-TOF studies. These findings align with the previous literature on factors affecting PMF quality, such as colony material, culture age, and sample preparation [25,26,27,28,29,30].

### 4.2. Sample Storage Effects on Spectral Consistency and Dataset Selection

We employed three datasets (T_1_, T_2_, and T_3_) derived from the same isolate collection. T_1_ and T_3_ contained PMF data from freshly cultured isolates, whereas T_2_ was generated from extracts stored for one year at −20 °C. PCA showed that T_2_ exhibited greater variability, likely due to protein degradation or handling effects. Removing T_2_ improved similarity across replicates, so T_2_ was excluded from primary analyses to ensure model training with consistent data, consistent with earlier reports that long-term storage can degrade protein profiles [29].

### 4.3. Preprocessing Challenges: Peak Alignment, Variability, and Information Loss

Preprocessing of raw MALDI-TOF data presented challenges that influenced downstream analyses. Biological and technical variability led to peak shifts that were difficult to correct, requiring careful calibration to generate reliable feature matrices. We developed similarity matrices to enable one-to-one comparisons, refining peak alignment while minimizing distortion of biologically meaningful features. Excessive correction risks removing minority peaks critical for classification [31], underscoring the need to balance correction with feature preservation.

The counter-intuitive finding that non-aligned T_2_ spectra showed higher similarity scores than aligned T_2_ spectra can be explained by an alignment overshoot artefact. The LOWESS warping function identifies stable reference peaks from a consensus spectrum derived predominantly from T_1_/T_3_ (fresh) spectra. When T_2_ spectra—which exhibit storage-induced peak broadening and *m*/*z* shifts—are warped to this fresh spectrum reference, the correction overshoots, distorting T_2_ peaks beyond their original positions and reducing intra-isolate concordance. This interpretation is supported by the observation that excluding T_2_ from the alignment reference (T_1+3_ only) improved alignment quality, with median intra-isolate similarity increasing to 97.7% (T_1_) and 97.2% (T_3_) in the aligned dataset versus 94.6% and 94.9% without alignment. From a clinical utility perspective, this finding indicates that MALDI-TOF ML pipelines should be trained on freshly prepared extracts; frozen-extract spectra should not be incorporated without separate alignment calibration.

### 4.4. Model Training Dataset Optimization and Performance Trends

After addressing alignment, we compared model performance across dataset configurations. The baseline dataset (T_1+3_) provided the best balance of phenotypic diversity and spectral stability. Including T_2_ (T_1+2+3_) increased recall for some antibiotics but often reduced precision, reflecting variability. Using non-aligned data (T_1+3_–NALI) increased FN counts, lowering recall. Thus, T_1+3_ was prioritized for primary evaluation as the most reliable training set.

### 4.5. Confusion Matrix Insights and Antibiotic-Specific Model Behavior

Confusion matrices revealed antibiotic-specific patterns. Ciprofloxacin showed the most balanced classification, with moderate recall (0.659) and precision (0.455), and the highest MCC (0.375). Amoxicillin achieved high recall (0.984) at the expense of precision (0.615), reflecting the threshold optimization favoring minority class detection. In contrast, amikacin and cefepime showed near-zero MCC values (0.023 and −0.001), confirming that apparent accuracy (>70%) was driven entirely by majority class dominance rather than genuine discrimination. Overall, the T_1+3_ ALI dataset produced the most consistent results, although sensitivity for resistant isolates remained limited for antibiotics with severe class imbalance.

### 4.6. Evaluating Classifier Performance: AUROC, AUPRC, and Metric Selection

Given the strong class imbalance in our isolate collection (resistant phenotype < 20% or >80%), we reported both AUROC and AUPRC as primary metrics. AUROC facilitated comparisons across antibiotics with different class ratios, but it can overestimate performance by ignoring precision. AUPRC complemented AUROC by focusing on the minority class, offering a more sensitive measure of resistant isolate detection. We additionally reported the Matthews Correlation Coefficient (MCC) and Cohen’s Kappa, which provide balanced measures that account for all four confusion matrix cells and are less susceptible to inflation by class imbalance than accuracy or F1-score. Bootstrap 95% confidence intervals (1000 replicates) were computed for all primary metrics to quantify estimation uncertainty. Together, this multi-metric framework provided a nuanced and robust view of model behavior across antibiotics with widely varying class distributions.

### 4.7. Performance Summary Across Antibiotics and Resistance Profiles

Across the 11 modeled antibiotics, the primary RF model achieved cross-validated F1-scores ranging from 0.057 (amikacin) to 0.757 (amoxicillin) and AUROC scores from 0.473 (cefepime) to 0.759 (ciprofloxacin). MCC ranged from −0.001 (cefepime) to 0.375 (ciprofloxacin). Only ciprofloxacin achieved an AUROC in the ‘fair’ range (0.759 [95% CI: 0.742–0.775]) [24]. Ceftazidime approached the ‘fair’ threshold (0.679 [0.649–0.711]). The remaining antibiotics showed AUROC values between 0.473 and 0.583, indicating ‘poor’ or ‘chance-level’ performance [24]. These cross-validated estimates are lower than those from our preliminary single-split analysis, reflecting the more conservative evaluation strategy: isolate-level grouping prevents optimistic bias from correlated technical replicates, and nested cross-validation prevents hyperparameter overfitting.

Despite threshold optimization (classification boundary set to maximize F1 on the precision–recall curve) and cost-sensitive class weighting (balanced_subsample), several antibiotics with extreme class imbalance still showed poor minority class detection. Amikacin (optimal threshold 0.399) and cefepime (optimal threshold 0.100) demonstrated that even with shifted decision boundaries, the lack of discriminative spectral features prevented meaningful classification. Ciprofloxacin performed best, achieving an AUROC of 0.759 and an AUPRC of 0.477, with the most balanced precision–recall trade-off among all antibiotics.

### 4.8. Comparison with External Studies and Literature Benchmarks

Our findings parallel external reports. Weis et al. (2022) applied ML to >300,000 PMFs across 803 bacterial and fungal species with >700,000 AMR labels, reporting AUROCs of 0.76 (ciprofloxacin), 0.73 (cefepime), and 0.60 (piperacillin–tazobactam) for *E. coli* [9]. Our cross-validated ciprofloxacin AUROC (0.759 [0.742–0.775]) is comparable to Weis et al.’s estimate of 0.76, while cefepime (0.473) and piperacillin–tazobactam (0.508) remained difficult to predict in both studies. Similarly, tobramycin showed poor performance in both studies (AUROC 0.546 here vs. 0.64 in Weis et al.). Importantly, direct comparison requires caution: our cross-validated, isolate-level estimates are inherently more conservative than single-split evaluations, and dataset sizes differ by two orders of magnitude (4468 spectra vs. >300,000). Additionally, Weis et al. used LightGBM and MLP classifiers, whereas we compared RF, LR, SVM, and GB—with McNemar’s test confirming that RF significantly outperformed LR for all antibiotics (*p* < 0.01), consistent with the observation that ensemble methods are better suited for high-dimensional spectral data.

Overall, both studies converge on a key insight: while machine learning models can perform well for certain resistance phenotypes (e.g., ciprofloxacin, ceftazidime), others remain difficult to classify accurately due to biological variability, class imbalance, or absence of detectable resistance markers in the MALDI-TOF mass range. These findings underscore the importance of antibiotic-specific model validation and cautious interpretation of performance metrics when applying MALDI-TOF MS-based resistance prediction in clinical contexts.

Nguyen et al. (2024) reported AUROC scores of 0.47–0.87 for 11 antibiotics in *P. aeruginosa* and demonstrated improved results when combining MALDI-TOF with genomic data or using dynamic binning [10]. These comparisons suggest that resistance to some antibiotics (e.g., ciprofloxacin, colistin) is more predictable than others (e.g., cefepime, aminoglycosides) and highlight the influence of dataset size, diversity, and preprocessing choices [10].

### 4.9. Clinical Risk Considerations and Model Sensitivity Trade-Offs

The acceptable performance of diagnostic models depends on the clinical context. In high-risk scenarios—such as infections in immunocompromised patients or those involving multidrug-resistant pathogens—high sensitivity is paramount to avoid FNs that could compromise therapy. In contrast, for lower-risk infections, the clinical value of moderately performing models is limited; empirical treatment with routine AST remains the safest approach. Models with intermediate accuracy may instead be useful in surveillance, outbreak monitoring, or as adjunctive decision support tools.

Ultimately, model development should emphasize robustness and generalizability, with performance thresholds aligned to the clinical consequences of prediction errors. Importantly, some antibiotics may inherently show lower predictive performance because resistance may not be detectable within the evaluated MALDI-TOF mass range (2–15 kDa). This limitation reflects biological constraints rather than algorithmic deficiencies.

Our findings have specific clinical implications. First, ciprofloxacin resistance is the most reliably predicted from MALDI-TOF spectra in our dataset, which could support rapid screening workflows in urinary tract infection management, where fluoroquinolones are frequently prescribed empirically. Second, the finding that frozen extract storage degrades model performance has direct implications for laboratory workflows: clinical MALDI-TOF ML systems should be trained and deployed on freshly prepared extracts. Third, the observation that certain antibiotics (amikacin, cefepime) show no predictive signal despite threshold optimization and cost-sensitive weighting highlights important limitations of MALDI-TOF-based AMR prediction for these resistance phenotypes in the present dataset, guiding future research toward complementary approaches (e.g., Raman spectroscopy, genomic data integration). Fourth, our new pipeline and complete code availability enable other laboratories to benchmark ML-based AMR prediction using their local strain collections.

## 5. Limitations and Future Perspectives

This study was limited by the size of the isolate collection (282 *E. coli* isolates, 4468 spectra). While modest for ML applications, our isolate-level cross-validation (GroupShuffleSplit) ensured that performance estimates reflect generalization to unseen isolates rather than unseen replicates. The concordance between GroupShuffleSplit and StratifiedGroupKFold results (e.g., ciprofloxacin F1: 0.538 vs. 0.527) supports the stability of our estimates. Nevertheless, expanding the collection—particularly for antibiotics with extreme class imbalance (amikacin, cefepime)—is a priority.

External validation was not performed due to the absence of publicly available *E. coli* MALDI-TOF datasets with matched MIC-based susceptibility profiles acquired using comparable instruments and protocols. We prioritized methodological transparency—publishing all code, parameters, and preprocessing pipelines—to enable external groups to reproduce and validate our results using their local strain collections.

Some antibiotics with low predictive performance may require additional investigation to clarify whether limitations arise from biological complexity, insufficient training data, or the absence of detectable resistance markers in the 2–15 kDa range. Integrating complementary data types (e.g., Raman spectroscopy, genomics) may enrich the feature space [10]. More advanced ML architectures (e.g., deep learning, ensemble stacking) and scalable preprocessing strategies are needed to enable real-time implementation in clinical workflows.

Our preprocessing relied on dynamic binning, which requires complete reprocessing whenever new data are added. While this approach effectively reduces dimensionality, it increases computational demands and limits scalability in real-time clinical workflows. Fixed binning options, which were not available in the ‘MALDIquant’ package [15], or alternative approaches may overcome these challenges [32]. Previous studies have also noted drawbacks of binning, including sensitivity to outliers and uncertainty in defining optimal bin sizes [33,34]. Despite these limitations, binning remains a widely used strategy for MALDI-TOF MS data, provided spectra are well aligned.

## 6. Conclusions

In this study, we successfully developed and evaluated a reproducible machine learning workflow for predicting antimicrobial resistance in *E. coli* from MALDI-TOF mass spectrometry data. The primary objective of establishing a transparent, standardized, and reproducible framework for MALDI-TOF-based antimicrobial resistance prediction was achieved through the implementation of an open-source preprocessing pipeline, rigorous isolate-level nested cross-validation, and systematic comparison of multiple machine learning algorithms.

Among the evaluated antibiotics, ciprofloxacin showed the strongest predictive performance, followed by ceftazidime, indicating that reproducible resistance-associated spectral signatures can be detected for selected resistance phenotypes. In contrast, amikacin, cefepime, tigecycline, piperacillin–tazobactam, and tobramycin exhibited near-random classification performance, suggesting that either insufficient resistance-associated spectral information is present within the investigated mass range or that larger datasets are required to capture more subtle resistance signatures.

The study also demonstrated that long-term storage of protein extracts at −20 °C negatively affected spectral reproducibility and introduced variability that reduced suitability for machine learning model development. Consequently, freshly prepared extracts or newly cultured isolates should be preferred for future MALDI-TOF-based machine learning workflows.

The comparison of machine learning algorithms showed that Random Forest and Gradient Boosting consistently outperformed linear approaches, supporting the importance of modeling non-linear relationships within MALDI-TOF spectral data. The combination of standardized preprocessing, feature selection, statistical model comparison, and transparent reporting provides a reproducible foundation for future development and external validation of MALDI-TOF-based antimicrobial resistance prediction systems.

Future studies should focus on larger multicenter datasets, external validation across laboratories, and integration with complementary genomic or phenotypic information to improve predictive performance and facilitate clinical implementation.

## Figures and Tables

**Table 1:** Overview of the different MALDI-TOF datasets used in the study. All data were acquired on a “Sirius” MALDI-TOF mass spectrometer.

Dataset Alias	Info	Site of MALDI-TOF Analysis	Date of PMF Generation
‘T_1_’	Protein extracts derived from fresh subcultures of *E. coli* isolates Total number of *E. coli* PMFs: 2228 (279 isolates)	University Hospital of Brussels	January 2022
‘T_2_’	Protein extracts stored at −20 °C for one year, originally prepared from the same ‘T_1_’ subcultures Total number of *E. coli* PMFs: 2224 (279 isolates)	Ghent University Hospital	January 2023
‘T_3_’	Protein extracts derived from new subcultures of the original *E. coli* isolates (biological replicates) Total number of *E. coli* PMFs: 2256 (282 isolates)	Ghent University Hospital	January 2023

**Table 2:** Overview of evaluation metrics used for binary classification performance assessment. TP = true positive (resistant correctly classified); TN = true negative (susceptible correctly classified); FP = false positive (susceptible misclassified as resistant); FN = false negative (resistant misclassified as susceptible); TPR = true positive rate; FPR = false positive rate.

Metric	Definition	Range	Interpretation
Accuracy	Proportion of all correctly classified spectra (TP + TN) out of the total	0–1	Overall correctness; misleading under class imbalance
Precision	Proportion of predicted resistant spectra that are truly resistant	0–1	Low FP rate; high precision = few false alarms
Recall (Sensitivity)	Proportion of truly resistant spectra correctly identified	0–1	Low FN rate; high recall = few missed resistant cases
F1-score	Harmonic mean of precision and recall	0–1	Balances precision and recall; preferred over accuracy under imbalance
AUROC	Area under the ROC curve (TPR vs. FPR across all thresholds)	0–1	Threshold-independent discrimination; 0.5 = random, 1.0 = perfect
AUPRC	Area under the precision–recall curve across all thresholds	0–1	Focuses on minority (resistant) class detection; more informative than AUROC under severe imbalance
MCC	Matthews Correlation Coefficient; correlation between observed and predicted classifications using all four confusion matrix cells	−1 to +1	0 = random, +1 = perfect, −1 = total disagreement; unaffected by class imbalance
Cohen’s Kappa	Agreement between predicted and observed classifications corrected for chance agreement	−1 to +1	0 = chance-level agreement, 1 = perfect agreement

**Table 3:** Antibiotic resistance prediction performance of the primary antibiotics.

Antibiotic	#R/#Total	#Features	Accuracy	Precision	Recall	F1	AUROC [95% CI]	AUPRC	MCC
Ciprofloxacin	928/4468	13	0.738	0.455	0.659	0.538	0.759 [0.742–0.775]	0.477	0.375
Ceftazidime	432/4468	37	0.812	0.270	0.556	0.363	0.679 [0.649–0.711]	0.251	0.293
Amoxicillin	2672/4468	37	0.621	0.615	0.984	0.757	0.583 [0.564–0.600]	0.661	0.154
Amoxicillin–clavulanic acid	1360/4468	25	0.363	0.331	0.963	0.493	0.561 [0.542–0.580]	0.404	0.080
Sulfamethoxazole–trimethoprim	1344/4468	37	0.381	0.321	0.929	0.477	0.548 [0.522–0.550]	0.357	0.102
Aztreonam	448/4468	85	0.709	0.187	0.571	0.282	0.567 [0.537–0.598]	0.157	0.193

**Table 4:** Antibiotic resistance prediction with poor/chance-level prediction performance.

Antibiotic	#R/#Total	Ratio	#Features	F1	AUROC	MCC
Amikacin	96/4468	45.5:1	1	0.057	0.558	0.023
Cefepime	192/4468	22.3:1	1	0.103	0.473	−0.001
Tigecycline	288/4468	14.5:1	1	0.171	0.570	0.102
Piperacillin–tazobactam	528/4468	7.5:1	1	0.239	0.508	0.060
Tobramycin	504/4468	7.9:1	1	0.212	0.546	0.098

**Table 5:** Feature selection summary.

Antibiotic	Initial Features	Selected Features	% Removed	Task Complexity
Amikacin	121	1	99.2%	Single-peak
Cefepime	121	1	99.2%	Single-peak
Piperacillin–tazobactam	121	1	99.2%	Single-peak
Tigecycline	121	1	99.2%	Single-peak
Tobramycin	121	1	99.2%	Single-peak
Ciprofloxacin	121	13	89.3%	Multi-peak
Amoxicillin–clavulanic acid	121	25	79.3%	Multi-peak
Amoxicillin	121	37	69.4%	Multi-peak
Ceftazidime	121	37	69.4%	Multi-peak
Sulfamethoxazole–trimethoprim	121	37	69.4%	Multi-peak
Aztreonam	121	85	29.8%	Complex signature

**Table 6:** Multi-model comparison. Bold = highest AUROC per row. *p*-values from McNemar’s test. *** *p* < 0.001; ** *p* < 0.01. Note: These AUROC values come from the model comparison section of the run, where all 4 models were trained on the same RFECV features and evaluated on pooled outer folds. They differ slightly from the primary RF results in Table 3 because the primary pipeline uses per-fold threshold optimization, whereas the comparison uses a single evaluation pass.

Antibiotic	RF AUROC	LR AUROC	SVM AUROC	GB AUROC	RF vs. LR (*p*)	RF vs. GB (*p*)
Ciprofloxacin	**0.782**	0.574	0.730	0.775	<0.001 ***	0.174
Ceftazidime	0.742	0.624	0.669	**0.800**	<0.001 ***	0.281
Amoxicillin	**0.600**	0.426	0.460	0.555	<0.001 ***	<0.001 ***
Amoxicillin–clavulanic acid	**0.598**	0.528	0.597	0.589	<0.001 ***	<0.001 ***
Sulfamethoxazole–trimethoprim	**0.636**	0.606	0.619	0.620	0.007 **	0.560
Aztreonam	0.657	0.631	0.622	**0.686**	<0.001 ***	<0.001 ***
Amikacin	0.687	0.517	**0.706**	0.638	<0.001 ***	<0.001 ***
Cefepime	0.492	**0.707**	0.468	0.544	<0.001 ***	<0.001 ***
Tigecycline	0.602	0.425	**0.636**	0.557	<0.001 ***	<0.001 ***
Piperacillin–tazobactam	0.533	**0.548**	0.538	0.520	<0.001 ***	<0.001 ***
Tobramycin	0.511	0.536	**0.557**	0.517	<0.001 ***	0.105

## Data Availability

The raw data supporting the conclusions of this article will be made available by the authors on request.

## Supplementary Materials

The following supporting information can be downloaded at https://www.mdpi.com/article/10.3390/diagnostics16132103/s1: Table S1: Hyperparameters used in the MALDI-TOF preprocessing pipeline (MTPP) and the machine learning classification pipeline; Table S2: Descriptive statistics of the similarity percentages between replicate PMFs in each dataset and dataset combinations. Descriptive statistics of replicates in each dataset are indicated in blue. Descriptive statistics of replicates in combined datasets are indicated in green. ALI: aligned, NALI: not aligned, PMF: peptide mass fingerprint, MTPP: MALDI-TOF preprocessing pipeline; Figure S1: Violin–boxplots illustrating the similarity percentages of replicate PMFs for MTPP-processed datasets T1 and T3, comparing aligned (Panel A) and non-aligned (Panel B) conditions. Panels C and D depict the similarity percentages of replicate PMFs for the same datasets, with aligned (Panel C) and non-aligned (Panel D) conditions; Table S3: Evaluation metrics for the predictive performance of antibiotic resistance by a random forest classification model for different combinations of the datasets (T1, T2, and T3) and for aligned (‘ALI’) and non-aligned (‘NALI’) PMF data. TN, true negatives; FP, false positives; FN, false negatives; TP, true positives; Table S4: Brief overview of tested conditions for optimization of experimental procedures; Figure S2: Similarity matrices of the ANI–MALDI-TOF MS T1MC (Panel A) and ANI–MALDI-TOF MS T1BP (Panel B). The ANI similarity values are presented on the horizontal axes and the MALDI-TOF MS values on the vertical axes. Both matrices were ordered according to the ANI values; Table S5: Hyperparameter search spaces for the three comparator models used in RandomizedSearchCV (n_iter = 50, inner CV: GroupKFold, n_splits = 5). LR used max_iter = 2000; SVM used probability = True; Table S6: Representative optimal Random Forest hyperparameter configurations and number of RFECV-selected features for each antibiotic. Hyperparameters were optimized independently for each antibiotic using RandomizedSearchCV within the inner loop of the nested cross-validation framework. The table reports the representative optimal configuration identified during model development, together with the final number of features retained after recursive feature elimination with cross-validation (RFECV); Table S7: Pairwise McNemar’s χ^2^ test results for multi-model comparison. Six pairwise comparisons per antibiotic (RF = Random Forest, LR = Logistic Regression, SVM = Support Vector Machine, GB = Gradient Boosting). Significance: *** *p* < 0.001, * *p* < 0.05, ns = not significant. All tests performed on aggregated outer test-fold predictions (n = 4468 spectra). Key pattern: RF significantly outperformed LR for all 11 antibiotics (all *p* < 0.01). RF and GB performed comparably (ns) for ciprofloxacin, ceftazidime, sulfamethoxazole–trimethoprim, and tobramycin.
