# Supplementary material for: Machine Learning-Based Prediction of Antimicrobial Resistance in Escherichia coli from MALDI-TOF Mass Spectrometry Data
Source: Diagnostics (Basel). 2026 Jul 4;16(13):2103. doi: 10.3390/diagnostics16132103 (PMC13360082; doi:10.3390/diagnostics16132103)
Supplement: Supplementary file 1 [file diagnostics-16-02103-s001.zip › diagnostics-4140351-supplementary.pdf]

SUPPLEMENTARY INFORMATION

Table S1. Hyperparameters used in the MALDI-TOF preprocessing pipeline (MTPP) and the machine learning classification pipeline.

MALDI-TOF PREPROCESSING PIPELINE (MTPP)			
**Processing step**	**Method**	**Key parameters**	**Rationale for selection**
**Variance stabilization**	\`sqrt\`	—	Standard for mass spectrometry data
**Smoothing**	\`SavitzkyGolay\`	halfWindowSize = 10	Default; larger windows showed no added benefit
**Baseline correction**	\`SNIP\`	iterations = 100	Stable baseline; commonly used default
**Normalization**	\`TIC\`	—	Standard practice in MALDI-TOF processing
**Alignment**	\`lowess\`	halfWindowSize = 20<br>SNR = 2<br>tolerance = 0.2	Refined for best replicate similarity
**Peak detection**	\`MAD\`	halfWindowSize = 20<br>SNR = 3	Visual inspection and test spectra quality
**Peak binning and filtering**		minFrequency = 0.10<br>Tolerance = 0.002	Removed noise, preserved shared peaks
**Generation of the feature matrix**	—	—	—
**Generation of the PMF similarity matrix**	—	t = 0, t = 3<br>b = 0	—

MACHINE LEARNING CLASSIFICATION PIPELINE		
**Processing step**	**Key parameters**	**Rationale for selection**
**Outer evaluation**	GroupShuffleSplit(n_splits=5, test_size=0.2, random_state=42)	Isolate-level grouping prevents data leakage; 5 repeats for stable estimates
**Feature selection (RFECV)**	estimator=RF, step=0.1, cv=GroupKFold(n_splits=5), scoring='f1'	10 % feature removal per step; grouped inner CV; F1 for minority-class focus
**Hyperparameter tuning**	RandomizedSearchCV(n_iter=50), cv=GroupKFold(n_splits=5)	Nested tuning prevents hyperparameter overfitting
**RF search space**	n_estimators ∈ {100,300,500}<br>max_depth ∈ {None,20,30}<br>min_samples_leaf ∈ {1,3,5}<br>max_features ∈ {sqrt,log2,0.3}<br>class_weight ∈ {balanced, balanced_subsample}	Broad search covering capacity, regularization, and imbalance handling
**Comparator models**	Logistic Regression, SVM, Gradient Boosting; tuned with identical RandomizedSearchCV framework (see **Table S5** for search spaces)	Multi-model comparison validates RF as primary classifier
**Threshold optimization**	Threshold set to maximize F1 on training PR curve	Addresses class imbalance beyond default 0.5
**Bootstrap CI**	1,000 replicates, percentile method	Quantifies estimation uncertainty
**Statistical comparison**	McNemar's χ^2 test, 6 pairwise comparisons per antibiotic	Validates model choice with significance testing

Table S2. Descriptive statistics of the similarity percentages between replicate PMFs in each dataset and dataset combinations. Descriptive statistics of replicates in each dataset are indicated in blue. Descriptive statistics of replicates in combined datasets are indicated in green. ALI: aligned, NALI: not aligned, PMF: peptide mass fingerprint, MTPP: MALDI-TOF preprocessing pipeline.

Datasets included in MTPP	MTPP similarity \`t\` value	Compared dataset	Alignment	# PMF	Mean (%)	SD (%)	Min. (%)	Q1 (%)	Median (%)	Q3 (%)	Max. (%)	Range (%)
T_{1+2+3}	3	T_1	ALI	7,822	80.50	24.29	2.64	58.62	95.64	97.91	99.76	2.64–99.76
	3	T_2	ALI	7,784	62.78	25.82	2.47	41.55	62.06	88.67	99.56	2.47–99.56
	3	T_3	ALI	7,904	86.20	21.10	4.17	87.45	96.47	97.85	99.78	4.17–99.78
	3	T_{1+2}	ALI	17,824	40.26	18.30	2.35	26.16	38.25	49.98	91.93	2.35–91.93
	3	T_{1+3}	ALI	17,888	73.43	25.37	1.93	54.07	87.37	92.93	98.96	1.93–98.96
	3	T_{2+3}	ALI	17,856	38.99	17.66	3.24	25.71	36.73	48.20	90.10	3.24–90.10
T_{1+2+3}	3	T_1	NALI	7,822	95.26	3.84	56.74	94.14	96.31	97.65	99.77	56.74–99.77
	3	T_2	NALI	7,784	88.23	11.91	29.05	86.53	92.89	95.88	99.37	29.05–99.37
	3	T_3	NALI	7,904	91.03	8.74	46.29	89.15	94.23	96.91	99.67	46.29–99.67
	3	T_{1+2}	NALI	17,824	65.29	13.46	24.22	55.59	69.92	75.29	93.10	24.22–93.10
	3	T_{1+3}	NALI	17,888	81.83	13.48	5.19	78.77	86.31	90.53	97.88	5.19–97.88
	3	T_{2+3}	NALI	17,856	64.40	13.44	10.47	54.39	69.18	74.51	88.63	10.47–88.63
T_{1+2+3}	0	T_1	ALI	7,822	80.50	24.29	2.64	58.62	95.64	97.91	99.76	2.64–99.76
	0	T_2	ALI	7,784	62.78	25.82	2.47	41.55	62.06	88.67	99.56	2.47–99.56
	0	T_3	ALI	7,904	86.20	21.10	4.17	87.45	96.47	97.85	99.78	4.17–99.78
	0	T_{1+2}	ALI	17,824	40.26	18.30	2.35	26.16	38.25	49.98	91.93	2.35–91.93
	0	T_{1+3}	ALI	17,888	73.43	25.37	1.93	54.07	87.37	92.93	98.96	1.93–98.96
	0	T_{2+3}	ALI	17,856	38.99	17.66	3.24	25.71	36.73	48.20	90.10	3.24–90.10
T_{1+2+3}	0	T_1	NALI	7,822	95.22	3.85	56.11	94.09	96.28	97.62	99.77	56.11–99.77
	0	T_2	NALI	7,784	87.22	12.32	29.03	85.19	92.06	95.32	99.18	29.03–99.18
	0	T_3	NALI	7,904	90.18	9.31	44.25	87.92	93.63	96.53	99.57	44.25–99.57
	0	T_{1+2}	NALI	17,824	64.88	13.64	23.82	54.89	69.60	75.01	92.98	23.82–92.98
	0	T_{1+3}	NALI	17,888	81.40	13.62	4.80	78.16	85.90	90.21	97.74	4.80–97.74
	0	T_{2+3}	NALI	17,856	63.67	13.80	10.47	53.03	68.67	74.11	88.20	10.47–88.20
T_{1+3}	3	T_1	ALI	7,822	96.55	5.36	21.79	96.46	97.70	98.54	99.76	21.79–99.76
	3	T_3	ALI	7,904	93.99	10.03	29.50	95.29	97.15	98.09	99.78	29.50–99.78
	3	T_{1+3}	ALI	17,888	88.99	11.17	10.74	88.67	92.15	94.34	98.94	10.74–98.94
T_{1+3}	3	T_1	NALI	7,822	92.65	6.21	47.79	89.92	94.59	97.07	99.70	47.79–99.70
	3	T_3	NALI	7,904	92.15	7.50	53.63	90.87	94.88	97.03	99.60	53.63–99.60
	3	T_{1+3}	NALI	17,888	81.23	12.85	9.51	77.78	85.12	89.45	97.83	9.51–97.83

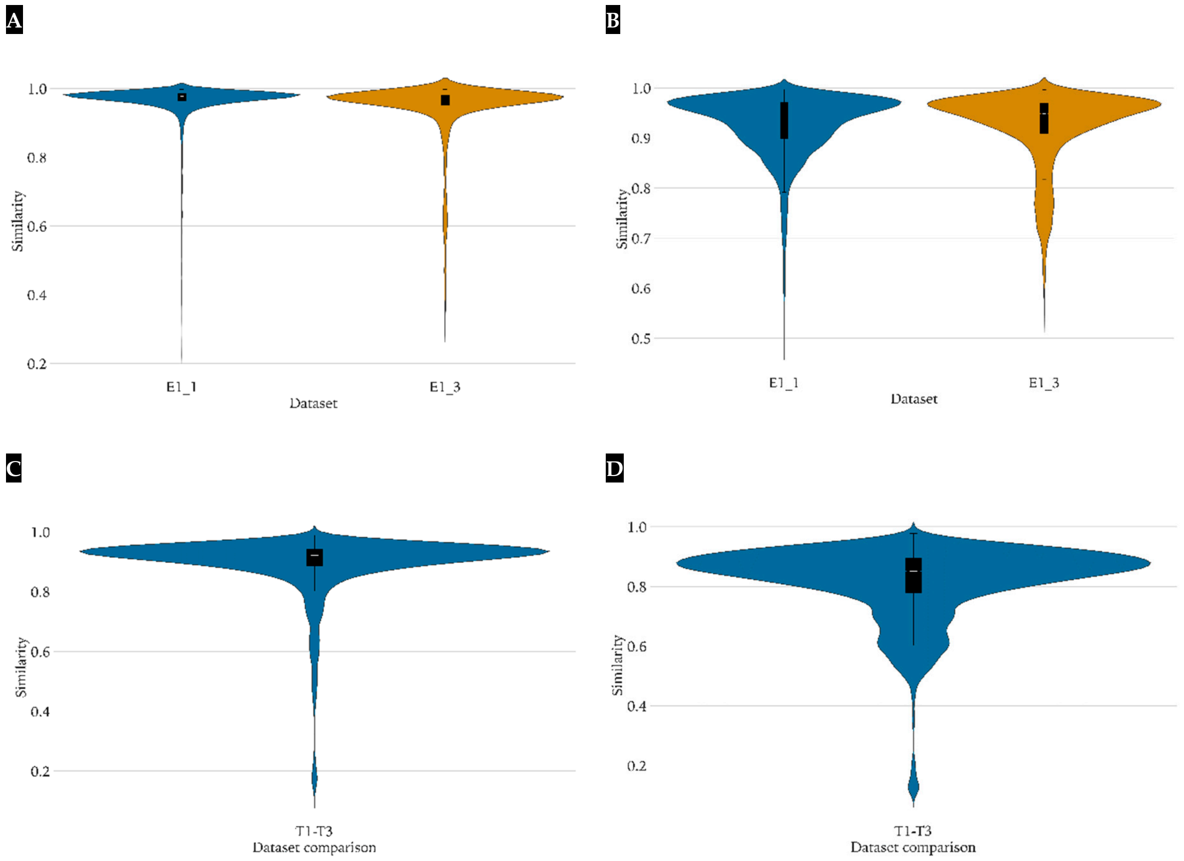

Figure S1. Violin–boxplots illustrating the similarity percentages of replicate PMFs for MTPP-processed datasets T_1 and T_3, comparing aligned (**Panel A**) and non-aligned (**Panel B**) conditions. Panels C and D depict the similarity percentages of replicate PMFs for the same datasets, with aligned (**Panel C**) and non-aligned (**Panel D**) conditions.

Table S3. Evaluation metrics for the predictive performance of antibiotic resistance by a random forest classification model for different combinations of the datasets (T_1, T_2, and T_3) and for aligned (`ALI`) and non-aligned (`NALI`) PMF data. TN, true negatives; FP, false positives; FN, false negatives; TP, true positives.

	Column	TN	FP	FN	TP	Accuracy	Precision	Recall	F1-Score	AUROC	AUPRC
T_1+T_3 – NALI	*Amikacin*	874	6	16	0	97.54%	0.00%	0.00%	0.00%	0.5015	0.0168
	Amoxicillin	168	88	294	346	57.37%	79.72%	54.06%	64.43%	0.6141	0.7887
	Amoxicillin–Clavulanic acid	530	46	246	74	67.41%	61.67%	23.13%	33.64%	0.6050	0.5185
	Aztreonam	719	17	141	19	82.37%	52.78%	11.88%	19.39%	0.6925	0.4180
	Cefepime	784	0	112	0	87.50%	0.00%	0.00%	0.00%	0.5223	0.1580
	Ceftazidime	717	3	144	32	83.59%	91.43%	18.18%	30.33%	0.7547	0.6039
	Ciprofloxacin	625	15	172	84	79.13%	84.85%	32.81%	47.32%	0.8551	0.7428
	Colistin	880	0	12	4	98.66%	100.00%	25.00%	40.00%	0.9986	0.9454
	Piperacillin–Tazobactam	727	9	148	12	82.48%	57.14%	7.50%	13.26%	0.6665	0.3831
	Tigecycline	799	1	92	4	89.62%	80.00%	4.17%	7.92%	0.6439	0.2782
	Tobramycin	768	16	101	11	86.94%	40.74%	9.82%	15.83%	0.6797	0.2804
	Sulfamethoxazole–trimethoprim	563	45	230	58	69.31%	56.31%	20.14%	29.67%	0.6018	0.4321
$T_1+T_2+T_3$ – ALI	*Amikacin*	1303	17	24	0	96.95%	0.00%	0.00%	0.00%	0.4254	0.0089
	Amoxicillin	254	130	443	517	57.37%	79.91%	53.85%	64.34%	0.6173	0.8018
	Amoxicillin–Clavulanic acid	827	37	386	94	68.53%	71.76%	19.58%	30.77%	0.6202	0.5487
	Aztreonam	1097	7	213	27	83.63%	79.41%	11.25%	19.71%	0.6083	0.3556
	Cefepime	1176	0	168	0	87.50%	0.00%	0.00%	0.00%	0.4619	0.0964
	Ceftazidime	1078	2	213	51	84.00%	96.23%	19.32%	32.18%	0.6752	0.5344
	Ciprofloxacin	947	13	304	80	76.41%	86.02%	20.83%	33.54%	0.7736	0.6413
	Colistin	1320	0	20	4	98.51%	100.00%	16.67%	28.57%	0.9783	0.9363
	Piperacillin–Tazobactam	1100	4	223	17	83.11%	80.95%	7.08%	13.03%	0.6620	0.3865
	Tigecycline	1199	1	141	3	89.43%	75.00%	2.08%	4.05%	0.6301	0.2430
	Tobramycin	1152	24	144	24	87.50%	50.00%	14.29%	22.22%	0.6955	0.3287
	Sulfamethoxazole–trimethoprim	833	79	324	108	70.01%	57.75%	25.00%	34.89%	0.6480	0.5030

$T_1+T_2+T_3$ – NALI	***Amikacin***	1301	19	24	0	96.80%	0.00%	0.00%	0.00%	0.4402	0.0089
	Amoxicillin	244	140	453	507	55.88%	78.36%	52.81%	63.10%	0.6170	0.8144
	Amoxicillin–Clavulanic acid	799	65	395	85	65.77%	56.67%	17.71%	26.98%	0.5620	0.4750
	Aztreonam	1087	17	212	28	82.96%	62.22%	11.67%	19.65%	0.6524	0.3989
	Cefepime	1176	0	168	0	87.50%	0.00%	0.00%	0.00%	0.4692	0.1043
	Ceftazidime	1076	4	224	40	83.04%	90.91%	15.15%	25.97%	0.7190	0.5869
	Ciprofloxacin	933	27	316	68	74.48%	71.58%	17.71%	28.39%	0.7984	0.6252
	Colistin	1320	0	14	10	98.96%	100.00%	41.67%	58.82%	0.9158	0.8715
	Piperacillin–Tazobactam	1095	9	220	20	82.96%	68.97%	8.33%	14.87%	0.6570	0.3704
	Tigecycline	1199	1	140	4	89.51%	80.00%	2.78%	5.37%	0.6407	0.2348
	Tobramycin	1152	24	139	29	87.87%	54.72%	17.26%	26.24%	0.6818	0.3521
	Sulfamethoxazole–trimethoprim	856	56	339	93	70.61%	62.42%	21.53%	32.01%	0.6595	0.5039

0

Table S4. Brief overview of tested conditions for optimization of experimental procedures.

Tested condition	Evaluation
**Influence of the culture growth medium on PMF characterization. TSA+5% sheep blood medium vs. MacConkey medium**	The similarity matrices revealed noticeable differences between Average Nucleotide Identity (ANI)–MALDI-TOF MS clustering patterns obtained from cultures grown on MacConkey medium versus those from TSA + 5 % sheep blood (SB). A stronger apparent concordance between ANI-based clustering and MALDI-TOF MS clustering was observed for isolates cultured on MacConkey medium. This observation is based on visual inspection of dendrogram structures rather than on formal statistical testing. [8]
**Reproducibility of PMF acquisition in time**	The similarity scores between PMFs of isolates cultured on MacConkey medium and between three measurements (T_0, $T_{0+2\ months}$, $T_{0+4\ months}$) showed that over the course of 4 months, the PMFs were very reproducible with only minor overall differences ($\Delta \leq 2.5$ %) (T_0: 100 %, $T_{0+2\ months}$: 98.5 %, $T_{0+4\ months}$: 97.5 %) compared to a $\Delta > 5.6$ % for TSA+5% SB cultures (T_0: 100 %, $T_{0+2\ months}$: 94.4 %, $T_{0+4\ months}$: not measured).
**Influence of the culture incubation time on PMF number of peaks**	The number of peptide peaks in PMFs varied based on incubation time and culture medium for two *E. coli* isolates grown on TSA + 5 % sheep blood and MacConkey agar. Overall, MacConkey cultures showed a broader peak range (58–123) compared to TSA + 5% SB (73–98). Peak counts increased notably between 14 and 16 hours for both media, with maxima observed at 18 hours (MacConkey) and 22 hours (TSA + 5 % SB). Beyond these times, peak numbers declined, especially at 24 hours. These results highlight incubation time as a key factor influencing PMF complexity, with optimal peptide detection occurring before 24 hours.
**Determination of the PMF mass range (m/z)**	For *E. coli* isolates cultured on MacConkey and TSA + 5 % sheep blood media, no peptide peaks were detected above 12 kDa and 13 kDa m/z, respectively. Nearly all peaks were found within the 2–10 kDa range, with a strong concentration between 2–6 kDa. As no peaks were observed above 13 kDa, further analyses were performed between 2–15 kDa (incl. margin of tolerance).

A **B**

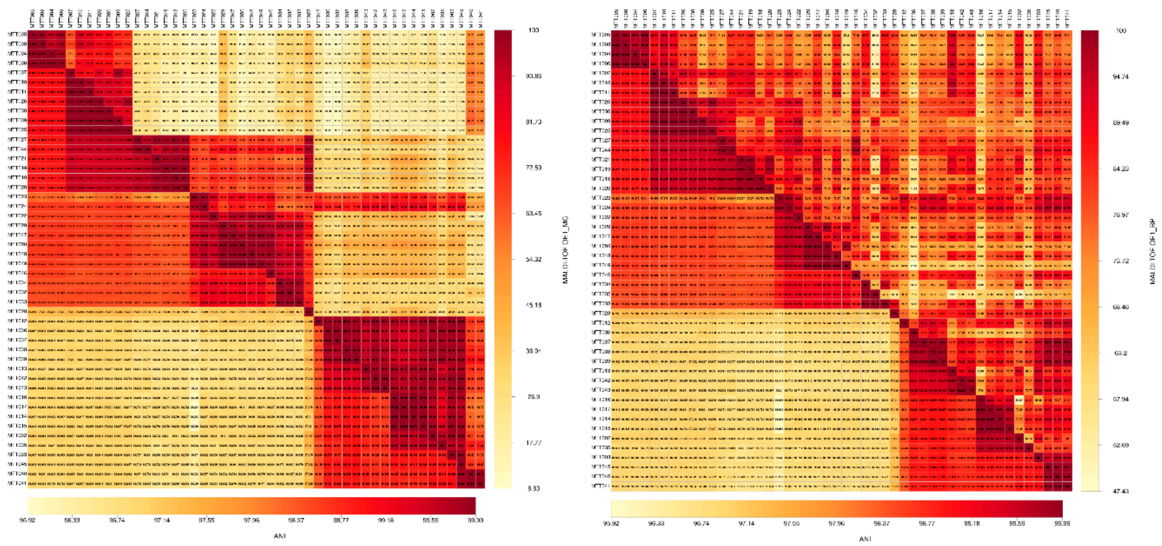

Figure S2. Similarity matrices of the ANI–MALDI-TOF MS T_1MC (**Panel A**) and ANI–MALDI-TOF MS T_1BP (**Panel B**). The ANI similarity values are presented on the horizontal axes and the MALDI-TOF MS values on the vertical axes. Both matrices were ordered according to the ANI values.

Table S5. Hyperparameter search spaces for the three comparator models used in RandomizedSearchCV (n_iter = 50, inner CV: GroupKFold, n_splits = 5). LR used max_iter = 2,000; SVM used probability = True.

Model	Hyperparameter	Search space
**Logistic Regression**	C	Uniform(0.01, 100)
	penalty	{l2}
	solver	{lbfgs, saga}
	class_weight	{balanced, None}
**SVM**	C	Uniform(0.1, 100)
	kernel	{rbf, linear}
	gamma	{scale, auto}
	class_weight	{balanced, None}
**Gradient Boosting**	n_estimators	{100, 300, 500}
	max_depth	{3, 5, 7}
	learning_rate	{0.01, 0.05, 0.1, 0.2}
	min_samples_leaf	{1, 3, 5}
	subsample	{0.8, 0.9, 1.0}

Table S6. Representative optimal Random Forest hyperparameter configurations and number of RFECV-selected features for each antibiotic. Hyperparameters were optimized independently for each antibiotic using RandomizedSearchCV within the inner loop of the nested cross-validation framework. The table reports the representative optimal configuration identified during model development, together with the final number of features retained after recursive feature elimination with cross-validation (RFECV).

Antibiotic	n_estimators	max_depth	max_features	min_samples_leaf	class_weight	n_features_selected
Amikacin	100	20	0.3	5	balanced	1
Amoxicillin	300	30	log2	1	balanced_subsample	37
Amoxicillin–clavulanic acid	100	None	0.3	5	balanced	25
Aztreonam	100	None	log2	5	balanced_subsample	85
Cefepime	100	20	0.3	5	balanced_subsample	1
Ceftazidime	500	None	log2	5	balanced_subsample	37
Ciprofloxacin	500	30	sqrt	5	balanced_subsample	13
Piperacillin-Tazobactam	500	None	log2	5	balanced_subsample	1
Tigecycline	100	20	sqrt	3	balanced	1
Tobramycin	100	20	0.3	5	balanced_subsample	1
Trimethoprim-Sulfamethoxazole	100	20	0.3	5	balanced_subsample	37

Table S7. Pairwise McNemar's χ^2 test results for multi-model comparison. Six pairwise comparisons per antibiotic (RF = Random Forest, LR = Logistic Regression, SVM = Support Vector Machine, GB = Gradient Boosting). Significance: *** p < 0.001, * p < 0.05, ns = not significant. All tests performed on aggregated outer test-fold predictions (n = 4,468 spectra).

Key pattern: RF significantly outperformed LR for all 11 antibiotics (all p < 0.01). RF and GB performed comparably (ns) for ciprofloxacin, ceftazidime, sulfamethoxazole–trimethoprim, and tobramycin.

Antibiotic	Comparison	χ^2	p-value	Sig.
**Amoxicillin**	RF vs LR	27.57	< 0.001	***
	RF vs SVM	15.03	< 0.001	***
	RF vs GB	36.39	< 0.001	***
	LR vs SVM	1.00	0.316	ns
	LR vs GB	0.20	0.659	ns
	SVM vs GB	2.73	0.099	ns
**Amoxicillin–clavulanic acid**	RF vs LR	21.25	< 0.001	***
	RF vs SVM	73.39	< 0.001	***
	RF vs GB	35.13	< 0.001	***
	LR vs SVM	12.25	< 0.001	***
	LR vs GB	0.86	0.353	ns
	SVM vs GB	19.23	< 0.001	***
**Amikacin**	RF vs LR	1019.05	< 0.001	***
	RF vs SVM	449.17	< 0.001	***
	RF vs GB	805.60	< 0.001	***
	LR vs SVM	389.02	< 0.001	***
	LR vs GB	67.99	< 0.001	***
	SVM vs GB	133.42	< 0.001	***
**Aztreonam**	RF vs LR	38.51	< 0.001	***
	RF vs SVM	59.29	< 0.001	***
	RF vs GB	38.47	< 0.001	***
	LR vs SVM	1.51	0.218	ns
	LR vs GB	0.30	0.584	ns
	SVM vs GB	4.17	0.041	*
**Ceftazidime**	RF vs LR	592.99	< 0.001	***
	RF vs SVM	8.42	0.004	*
	RF vs GB	1.16	0.281	ns
	LR vs SVM	647.73	< 0.001	***
	LR vs GB	603.98	< 0.001	***
	SVM vs GB	3.44	0.064	ns
**Ciprofloxacin**	RF vs LR	724.68	< 0.001	***
	RF vs SVM	6.89	0.009	*
	RF vs GB	1.84	0.174	ns
	LR vs SVM	654.41	< 0.001	***
	LR vs GB	730.31	< 0.001	***
	SVM vs GB	13.91	< 0.001	***

**Cefepime**	RF vs LR	593.19	< 0.001	***
	RF vs SVM	25.98	< 0.001	***
	RF vs GB	46.78	< 0.001	***
	LR vs SVM	1021.44	< 0.001	***
	LR vs GB	924.40	< 0.001	***
	SVM vs GB	0.84	0.361	ns
**Sulfamethoxazole–trimethoprim**	RF vs LR	7.17	0.007	*
	RF vs SVM	82.25	< 0.001	***
	RF vs GB	0.34	0.560	ns
	LR vs SVM	28.74	< 0.001	***
	LR vs GB	4.10	0.043	*
	SVM vs GB	73.75	< 0.001	***
**Tigecycline**	RF vs LR	14.44	< 0.001	***
	RF vs SVM	9.77	0.002	*
	RF vs GB	21.26	< 0.001	***
	LR vs SVM	0.50	0.480	ns
	LR vs GB	55.37	< 0.001	***
	SVM vs GB	49.17	< 0.001	***
**Tobramycin**	RF vs LR	59.94	< 0.001	***
	RF vs SVM	29.31	< 0.001	***
	RF vs GB	2.62	0.105	ns
	LR vs SVM	9.43	0.002	*
	LR vs GB	90.79	< 0.001	***
	SVM vs GB	74.53	< 0.001	***
**Piperacillin–tazobactam**	RF vs LR	366.65	< 0.001	***
	RF vs SVM	184.51	< 0.001	***
	RF vs GB	21.31	< 0.001	***
	LR vs SVM	57.57	< 0.001	***
	LR vs GB	244.13	< 0.001	***
	SVM vs GB	136.56	< 0.001	***
